# Sphingosine 1-Phosphate Induces Differentiation of Mesoangioblasts towards Smooth Muscle. A Role for GATA6

**DOI:** 10.1371/journal.pone.0020389

**Published:** 2011-05-24

**Authors:** Chiara Donati, Giuseppina Marseglia, Alberto Magi, Simona Serratì, Francesca Cencetti, Caterina Bernacchioni, Genni Nannetti, Matteo Benelli, Silvia Brunelli, Francesca Torricelli, Giulio Cossu, Paola Bruni

**Affiliations:** 1 Dipartimento di Scienze Biochimiche, Università di Firenze, Firenze, Italia; 2 Istituto Interuniversitario di Miologia (IIM), Italia; 3 SOD di Diagnostica Genetica, Azienda Ospedaliero Universitaria, Firenze, Italia; 4 Dipartimento di Oncologia e Patologia Sperimentali, Università di Firenze, Firenze, Italia; 5 Divisione di Medicina Rigenerativa, Istituto Scientifico H San Raffaele, Milano, Italia; 6 Dipartimento di Medicina Sperimentale, Università di Milano-Bicocca, Monza, Italia; 7 Dipartimento di Biologia, Università di Milano, Milano, Italia; Brigham & Women's Hospital - Harvard Medical School, United States of America

## Abstract

Different cells can contribute to repair following vascular injury by differentiating into smooth muscle (SM) cells; however the extracellular signals involved are presently poorly characterized. Mesoangioblasts are progenitor cells capable of differentiating into various mesoderm cell types including SM cells. In this study the biological action exerted by the pleiotropic sphingolipid sphingosine 1-phosphate (S1P) in human mesoangioblasts has been initially investigated by cDNA microarray analysis. Obtained data confirmed the anti-apoptotic action of this sphingolipid and identified for the first time a strong differentiating action toward SM cells. Quantitative mRNA and protein analysis corroborated the microarray results demonstrating enhanced expression of myogenic marker proteins and regulation of the expression of transcription factor GATA6 and its co-regulator, LMCD1. Importantly, GATA6 up-regulation induced by S1P was responsible for the enhanced expression of SM-specific contractile proteins. Moreover, by specific gene silencing experiments GATA6 was critical in the pro-differentiating activity of the cytokine TGFβ. Finally, the pharmacological inhibition of endogenous S1P formation in response to TGFβ abrogated GATA6 up-regulation, supporting the view that the S1P pathway plays a physiological role in mediating the pro-myogenic effect of TGFβ. This study individuates GATA6 as novel player in the complex transcriptional regulation of mesoangioblast differentiation into SM cells and highlights a role for S1P to favour vascular regeneration.

## Introduction

Smooth muscle (SM) cells control many fundamental functions such as arterial tone and airway resistance; alterations in vascular SM cells contribute to a number of diseases in humans including atherosclerosis and hypertension [1]. SM cells are not terminally differentiated and are able to switch between a contractile and synthetic phenotype in response to changing local environmental cues [2]. A large number of factors including mechanical forces, extracellular matrix components, endothelium-SM interactions and transforming growth factor-β (TGFβ) have been shown to promote SM marker gene expression in cultured cell systems [1]. However, in contrast to skeletal muscle development, no master genes have been found to regulate smooth muscle development, although several genes such as myocardin, MRTFA, MRTFB, Necdin and Msx2 have been found to be involved in the process [3], [4]. Several recent studies demonstrated that circulating, SM progenitor cells can contribute to neointima formation and repair following vascular injury [5].

Mesoangioblasts are a new type of progenitor cells, isolated from explants of dorsal aorta, capable of differentiating into various mesoderm cell types, such as smooth and striated muscle, bone and endothelium [6]. When delivered in the left ventricle, mesoangioblasts cause significant functional recovery despite modest anatomical repair of infarcted cardiac muscle [7].

The sphingolipid metabolite sphingosine 1-phosphate (S1P) is a lipid mediator that regulates fundamental biological processes mainly through binding to its specific receptors S1P_1–5_ in many cell systems [8]. S1P has recently been shown to have interesting effects on vascular development and SM cells growth and migration. S1P stimulates angiogenesis and induces vascular maturation in many experimental models [9]. Recent literature highlights the role of S1P in the regulation of proliferation, survival, differentiation and migration of a range of adult and embryonic stem cells [10]. We previously demonstrated that S1P acts as potent mitogen and anti-apoptotic agent in murine and human mesoangioblasts [11]. The important role of S1P in these cells, is further supported by our finding that the anti-apoptotic action of transforming growth factor β (TGFβ) involves the regulation of sphingosine kinase (SphK)1, critically implicated in S1P formation [12].

In order to completely utilize the therapeutic potential of these cells, it is important to understand their intrinsic properties and the role of the microenvironment in modulating their behaviour and function. To this end, to fully individuate the potential biological action of the pleiotropic cue S1P, we established transcriptional profiles of human mesoangioblasts treated with the bioactive sphingolipid. Presented results demonstrate that S1P promotes differentiation of human mesoangioblasts towards SM cells by transcriptional up-regulation of GATA6 and LMCD1. Moreover, we present evidence that TGFβ-induced differentiation of mesoangioblasts into SM relies on GATA6 and LMCD1 and that the induction of these two transcription factors depends on SphK/S1P axis.

## Methods

### Materials

Biochemicals, cell culture reagents, Dulbecco's modified Eagle's medium (DMEM), fetal calf serum (FCS), protease inhibitor cocktail, bovine serum albumin (BSA), Tetramethylrhodamine B isothiocyanate (TRITC)-phalloidin conjugate were purchased from Sigma (St. Louis, MO, USA). RNeasy Micro Kit was from Qiagen, (Valencia, CA, USA). TGFβ1 and basic fibroblast growth factor were purchased from PeproTech (London, UK). SKI-2 [2-(*p*-hydroxyanilino)-4-(*p*11chlorophenyl)thiazole] and D-*erythro*-S1P were from Calbiochem (San Diego, CA, USA). Enhanced chemiluminescence (ECL) reagents were obtained from GE Healthcare Europe GmbH (Milan, Italy). Coomassie Blue reagent was from Bio-Rad (Hercules, CA, USA). Secondary antibodies conjugated to horseradish peroxidase, anti-laminin, anti-calponin 1 (CNN1), anti-GATA6, anti-tropomyosin 1 (TPM1) antibodies, and non fat dry milk were obtained from Santa Cruz Biotechnology, Inc. (Santa Cruz, CA, USA). Goat anti-transgelin (TAGLN) antibodies was from Everest Biotech (Oxfordshire, OX, UK). Fluorescein-conjugated horse anti-mouse and anti-rabbit secondary antibodies were obtained from Vector (Burlingame, CA, USA). High Capacity cDNA Reverse Transcription Kit and all reagents and probes required to perform Real-Time PCR were from Applied Biosystems Inc. (Foster City, CA, USA). Two short interfering RNA (siRNA) duplexes corresponding to two DNA target sequences of human GATA6 (5′- GACAGAACGUGAUUCUCGUdTdT-3′ and 5′- GCUCAAGUAUUCGGGUCAAdTdT-3′), human LMCD1 (5′-GCCAUUACUGCGAGAGUCUdTdT-3′ and 5′-GGUCUACUCGGACAGGGCAdTdT-3′), one siRNA duplex corresponding to scrambled siRNA (5′-UUCUCCGAACGUGUCACGUdTdT-3′), Mission siRNA duplex corresponding to DNA target sequences of human SphK1 (SASI Hs01_00169403) and Mission siRNA duplex corresponding to DNA target sequences of human SphK2 (SASI Hs01_00158544) were purchased from Sigma-Proligo (The Woodlands, TX, USA). Lipofectamine RNAiMAX™ were purchased from Life Technologies (Carlsbad, CA, USA). Matrigel™ basement membrane matrix was purchased from BD Biosciences (Bedford, MA, USA).

The specific S1P_1/3_ antagonist, VPC23019 [13] and the S1P_2_ antagonist JTE23019 [14] were obtained from Tocris Cookson Ltd. (Bristol, UK). The ABCC1 inhibitor MK571 [15] was from Enzo Lifesciences, (Farmingdale, NY, USA).

### Cell culture

Human mesoangioblasts were routinely grown as previously described [16].

For SM differentiation experiments, routine culture medium on 90% confluent cells was changed to DMEM and incubated with 0.11 µmol/L TGFβ or 1 µmol/L S1P. In some experiments cells were treated with 10 µmol/L SKI-2, 1 µmol/L VPC23019 and 1 µmol/L JTE013 45 min before agonist challenge.

### Short interfering RNA administration

To silence SphK1, SphK2, GATA6 or LMCD1, mesoangioblasts were transfected with siRNA using a non specific siRNA as control. Cells grown into 6-well dishes (80,000 cells/well) in Mega Cell Medium supplemented with 5% heat inactivated FCS, were transfected with the 21-nucleotide duplexes using RNAiMAX™ Reagent according to manufacturer's instructions as previously demonstrated [17]. Briefly, RNAiMAX™ Reagent was incubated with siRNA (200 nM) at room temperature for 20 min and successively the lipid/RNA complexes were added with gentle agitation to the cells. After 48 h from the beginning of transfection cells were used for the experiments. To evaluate the specific knock-down of target genes, quantitative Real-Time PCR was performed.

### cDNA microarray

Agilent whole human genome oligonucleotide microarrays (44 K) were used to examine alterations in gene expression of cells after S1P treatment (Agilent Technologies, Santa Clara, CA). For the hybridization, 2 µg of total RNA were used. Labeling reaction, hybridization and washing of the array were performed following the protocols provided by Agilent according to the Two color microarray-based gene expression analysis. Arrays were scanned by using a 4000B Scanner (Axon Instruments, Union City, CA, USA). Each hybridization produced a 16-bit image, which was processed using the Agilent Feature Extraction software (v8.1). In order to identify differentially expressed genes, the data obtained from Feature Extraction were analyzed by “Significance Analysis of Microarray” (SAM) algorithm [18] using the R package siggenes.

All the statistical analyses were performed by using the statistical language R [19].

### Immunostaining and fluorescence microscopy

Cells were seeded on Lab-Tek II Chamber slides (Nalge Nunc International, Naperville, IL, USA), challenged with 1 µmol/L S1P or 0.11 µmol/L TGFβ for 48 h and processed as previously described [20]. Images were obtained using a Leica SP5 laser scanning confocal microscope with 40× and 63× objectives.

### Western blot analysis

Total cell lysates were prepared and subjected to SDS-PAGE electrophoresis and Western analysis as previously described [21].

### Quantitative Real-Time RT-PCR

Total RNA (2 µg), extracted with RNeasy Micro Kit from human mesoangioblasts, was reverse-transcribed using High Capacity cDNA Reverse Transcription Kit following the manufacturer's protocols. The quantification of target gene mRNAs was performed by Real-Time PCR employing TaqMan Gene Expression Assays. Simultaneous amplification of the target sequences (TPM1, Hs01576435_m1, CNN1, Hs00154543_m1, GATA6, Hs00232018_m1, TRPS1, Hs00232645_m1, LMCD1, Hs00205871_m1, Gene Expression assays, Applied Biosystems, Foster City, CA) together with the housekeeping gene, 18S rRNA, was carried out essentially as previously described [11]. The 2^−ΔΔCT^ method was applied as a comparative method of quantification [22] and data were normalized to ribosomal 18S RNA expression.

### *In vitro* morphogenesis assay

Matrigel (50 µl/well, 8–12 mg/ml) was pipetted into 13 mm tissue culture wells and polymerized for 30 min to 1 h at 37°C, as described [23]. Human mesoangioblasts were plated (20×10^3^/well) in Mega Cell Medium, supplemented with 2% FCS in the presence or absence of 1 µmol/L S1P or 10 µmol/L SKI-2. The effects on the morphological changes of mesoangioblats were recorded after 24 h with an inverted microscope (Leitz DM_IRB) equipped with CCD optics and a digital analysis system.

### Statistical analysis

Densitometric analysis of the Western Blot bands was performed using ImageJ and reported as percentage relative to the intensity of the band corresponding to control set as 100. Data are expressed as means ± S.E.M. of at least three experiments. Statistical analysis was performed using Student's t-test. Asterisks indicate statistical significance: *P<0.05.

## Results

A cDNA microarray study was performed to establish transcriptional profiles of human mesoangioblasts treated with the sphingolipid. For this purpose, when human mesoangioblasts were 90% confluent, the medium was changed to DMEM containing 0.1% BSA and further incubated in the presence of 1 µmol/L S1P for 6 h or 24 h.

**Figure 1:**
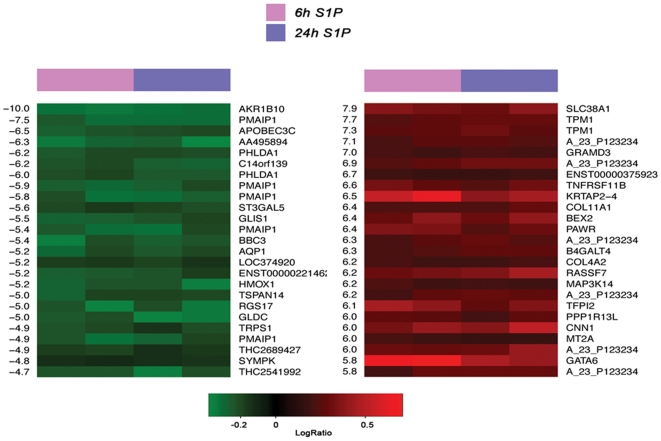
cDNA microarray analysis. The heat map shows the top 25 down-regulated (left, green) and top 25 up-regulated (right, red) genes. On the left and right side d-values and gene names are reported respectively. Columns 1–2 represent S1P stimulation for 6 h and columns 3–4 represent S1P stimulation for 24 h.

In our set of four arrays 369 up-regulated and 166 down-regulated genes were found to be significant (Supplemental table S1,S2). As shown in Fig. 1, after analysis of the top 25 up-regulated and top 25 down-regulated genes by S1P we found that genes involved in apoptosis induction such as *PMAIP1* and *BBC3* were significantly down-regulated. *PMAIP1 and BBC3* encode for Noxa and Puma respectively that belong to the BH3-only Bcl2 pro-apoptotic family of proteins [24]. On the contrary, but consistently with these results, the anti-apoptotic gene *PPP1R13L*, which encodes for a protein that blocks apoptosis by presumably binding and blocking p53, was found to be up-regulated following S1P challenge [25]. The S1P-dependent regulation of genes involved in apoptosis confirmed the anti-apoptotic role of this sphingolipid previously demonstrated in these progenitor cells [11].

Moreover, as shown in Fig. 1, S1P regulated the expression of genes involved in SM differentiation such as the transcription factors *GATA6* and *TRPS1* and those encoding for SM contractile proteins CNN1 and TPM1.

GATA6 is the only member of the GATA family transcription factors that is expressed in VSMC [26] and is rapidly down-regulated upon mitogen stimulation [27]. Although the downstream SM-specific genes targeted by GATA6 has yet to be completely identified, it plays a crucial role in the maintenance of the differentiated phenotype in VSMC [28]. Interestingly, in concert with S1P-induced up-regulation of GATA6 expression, *TRPS1* which encodes for a nuclear protein that binds GATA consensus motif and potently represses transcriptional activation mediated by other GATA factors [29], was among the genes maximally down-regulated following S1P treatment.

From the complete analysis of the microarray data (Supplemental tables S1,S2) emerged that the gene encoding for the LIM domain protein, LMCD1, was significantly up-regulated after S1P treatment. Expression of LMCD1 is remarkably similar to that of GATA6, with high level of expression observed in vascular SM cells and myocardium [30].

Notably, mRNA encoding for SM contractile apparatus proteins such as CNN1, that binds very strongly to actin in a Ca^2+^-independent manner, and the thin filament component TPM1 were among the most significantly up-regulated by S1P (Fig. 1). Furthermore, the expression of genes encoding for the SM-specific actin-binding protein caldesmon (CALD1) and TAGLN was also found to be significantly augmented (Supplemental table S1) supporting the view that not only S1P controls the transcriptional machinery which gives rise to the SM phenotype but S1P itself is also responsible for the onset of the differentiated phenotype. From the analysis of the transcriptional profile, Necdin and Msx2, previously identified as transcription factors required for SM differentiation of mesoangioblasts [3], [4], were not found to be modified by S1P treatment.

**Figure 2:**
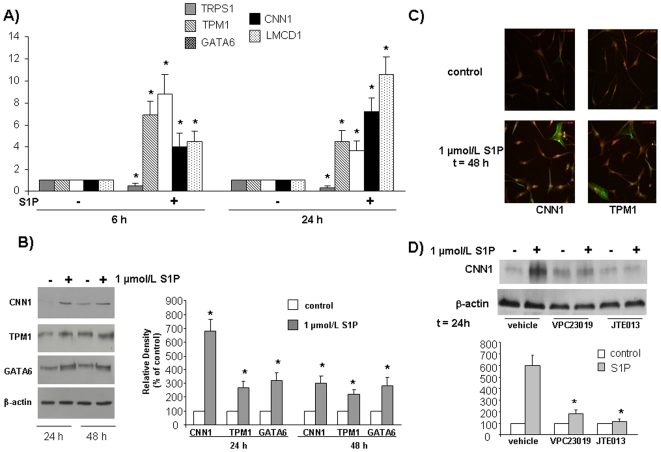
Effect of S1P on mRNA (A) and protein (B,C) expression levels of SM markers in human mesoangioblasts. Role of S1PR (D). Cells were incubated in DMEM with S1P and analyzed by A) Real-Time PCR; B) Western blot analysis; (lower panel represents densitometric quantification) and C) Confocal microscopy analysis (40× magnification) using TRITC-phalloidin and anti-fluorescein conjugated specific antibodies. D) Cells were pre-incubated with VPC23017 and JTE071 in DMEM before being challenged with S1P and analyzed by Western blot analysis for CNN1 (lower panel represents densitometric quantification).

Subsequently, in order to confirm the cDNA microarray findings, we performed a Real Time RT-PCR analysis of specific genes involved in SM differentiation that were differentially expressed following S1P challenge. Mesoangioblast treatment with 1 µmol/L S1P for 6 h and 24 h significantly increased mRNA levels of TPM1, GATA6, CNN1 and LMCD1, while it significantly decreased that of TRPS1. These results are consistent with that of the microarray data (Fig. 2A). The fold increase of TPM1 and GATA6 mRNA expression levels were higher at 6 h, while those of CNN1 and LMCD1 were higher at 24 h treatment with the sphingolipid, indicating a different time-course of expression for these genes. In agreement, GATA6 expression was also found more potently increased by S1P at 6 h than at 24 h in the cDNA microarray (Fig. 1).

Total lysates of serum-starved mesoangioblasts challenged with 1 µmol/L S1P for 24 h and 48 h were then analyzed by Western blot analysis for SM contractile apparatus proteins CNN1 and TPM1, and the transcription factor GATA6. S1P increased protein levels of all investigated markers after 24 h of incubation and its effect was still significant at 48 h (Fig. 2B). The morphological change provoked by this bioactive sphingolipid was clearly appreciable by confocal immunofluorescent microscopic analysis of mesoangioblasts performed to reveal the state of assembly of the cytoskeleton. Indeed, cell incubation with 1 µmol/L S1P for 48 h, profoundly altered the organization of actin cytoskeleton, giving rise to the formation of numerous F-actin filaments organized as stress fibers anchored to the plasma membrane, which were immunorevealed by TRITC-phalloidin (Fig. 2C). Notably, the same figure shows that a certain number of mesoangioblasts (approximately 30–40%) was strongly positive to the antibodies against CNN1 and TPM1 after S1P treatment. Thus, data presented in Figs. 2B and 2C demonstrate that the effect elicited by S1P on mesoangioblast protein levels was consistent with changes of the transcriptional profile induced by this sphingolipid.

The involvement of S1PR in the differentiating action of S1P was next examined. Interestingly, as shown in Fig. 2D, pre-incubation of human mesoangioblasts with the specific antagonist of S1P_1/3_ VPC23019 (1 µmol/L) or the specific antagonist of S1P_2_ JTE013 (1 µmol/L) completely abolished the increase of the SM marker CNN1 induced by 1 µmol/L S1P, suggesting that S1P_2_ and S1P_1/3_ receptors mediate distinct molecular events necessary for the differentiation to occur.

**Figure 3:**
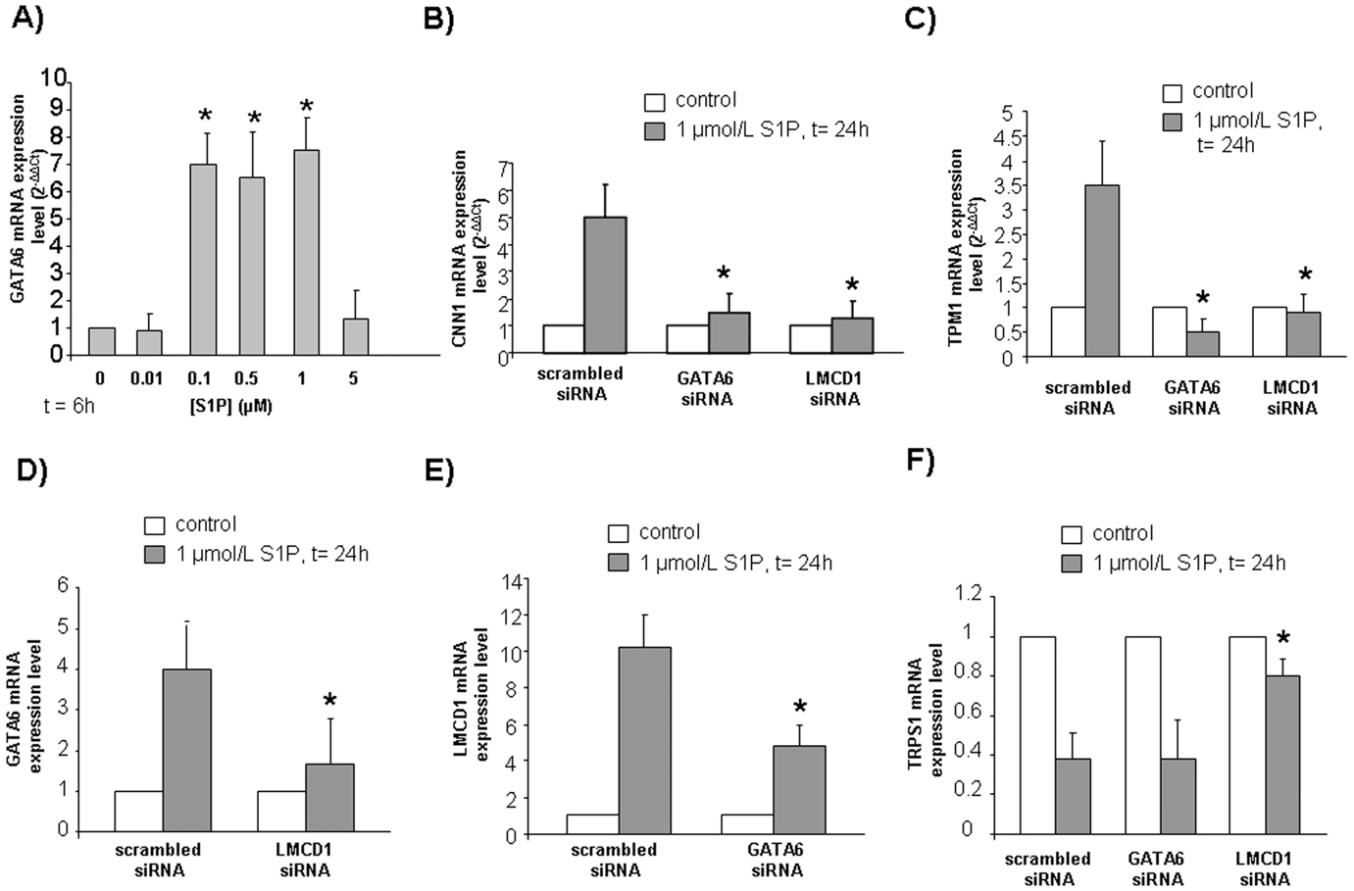
Dose-dependence of GATA6 expression level induced by S1P (A) and role of the transcription factors GATA6, LMCD1 (B,C) on the expression levels of SM markers induced by S1P in mesoangioblasts. Mutual regulation of the transcription factors GATA6, LMCD1 and TRPS1 (D,E,F). Real time PCR analysis of GATA6 (A) was performed in cells challenged with the indicated concentrations of S1P for 6 h. Real-Time PCR analysis of CNN1 (B), TPM1 (C), GATA6 (D), LMCD1 (E), TRPS1 (F) was performed in cells transfected with scrambled-, GATA6- or LMCD1-siRNA and stimulated with S1P for 24 h.

Given that GATA6 is recognized as a transcription factor critical for SM differentiation [31], to better characterize the increase of this transcription factor induced by S1P, we next determined if the sphingolipid augmented GATA6 mRNA expression levels in a dose-dependent manner. As shown in Fig. 3A, the sphingolipid was efficacious in upregulating GATA6 mRNA at a concentration of 100 nmol/L up to 1 µmol/L, while was ineffective at a higher concentration, in keeping with the affinity towards S1P exhibited by its receptors.

Moreover, we next investigated by Real Time PCR whether the up-regulation of the transcription factor GATA6 and LMCD1 mRNA content elicited by S1P was involved in the induction of the myogenic marker CNN1. As illustrated in Fig. 3B, GATA6 knock-down by specific RNA silencing almost abolished the S1P-dependent enhancement of CNN1 mRNA. The positive effect of S1P on the myogenic marker mRNA levels was also abolished by LMCD1 silencing. In perfect agreement, the down-regulation of GATA6 or LMCD1 mRNA abolished the S1P-induced increase of mRNA encoding for another key myogenic marker such as TPM1 (Fig. 3C).

To gain insight into the mechanism by which GATA6 and LMCD1 are necessary for the induction of expression of proteins characteristic of SM phenotype such as CNN1 and TPM1, their mRNA content in response to S1P administration was evaluated when one of the two transcriptional regulators was specifically silenced. Data reported in Fig. 3D show that GATA6 mRNA up-regulation induced by S1P was strongly reduced by LMCD1 silencing; likewise, knock-down of GATA6 was responsible for a markedly diminished effect of S1P on LMCD1 mRNA content (Fig. 3E). Thus, reciprocal regulation of GATA6 and LMCD1 mRNA levels accounts for the observed dual control of SM marker proteins by S1P.

The role of GATA6 and LMCD1 in the transcriptional regulation triggered by S1P in mesoangioblasts was further investigated by examining their involvement in the down-regulation of the GATA repressor TRPS1. Specific siRNA targeted to GATA6 did not influence the potent inhibitory effect of S1P on TRPS1 mRNA expression, whereas the efficacy of S1P was strongly attenuated when LMCD1 was silenced, clearly demonstrating that LMCD1 acted also independently from GATA6 regulation (Fig. 3F).

**Figure 4:**
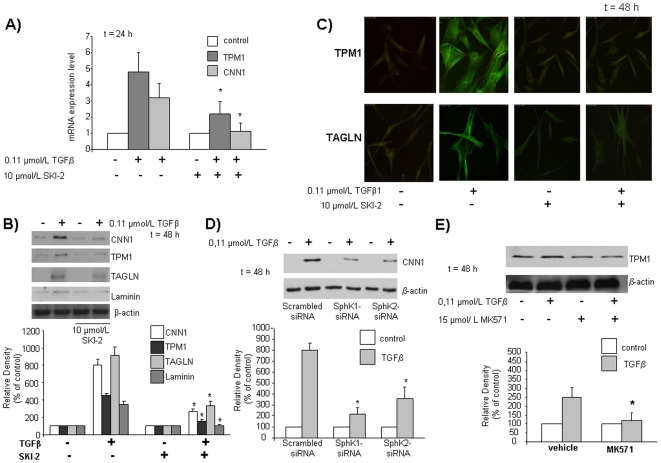
Effect of SphK inhibition (A,B,C), dowregulation (D) and ABCC1 transporter inhibition (E) on the expression levels of SM markers induced by TGFβ treatment in human mesoangioblasts. Cells were pre-treated with SKI-2 45 min before being incubated with DMEM for 48 h with TGFβ and analyzed by A) Real-Time PCR analysis B) Western blot analysis and C) Confocal microscopy analysis (63× magnification) using anti fluorescein-conjugated specific antibodies. D) Cells were transfected with scrambled-, SphK1-, SphK2-siRNA, stimulated with TGFβ and analyzed by Western blot analysis for CNN1. Lower panel represents densitometric quantification. E) Cells were pre-treated with MK571 45 min in DMEM before being incubated with TGFβ for 48 h and analyzed by Western blot analysis for TPM1. Lower panel represents densitometric quantification.

The current view indicates that a functional cross-talk between TGFβ and S1P signaling occurs in some cell types [32]–[35]. In this regard, we previously showed that TGFβ-dependent SphK/S1P axis is critical for the anti-apoptotic effect of the cytokine in mesoangioblasts [12]. Therefore, since TGFβ is a strong inducer of mesoangioblast differentiation towards SM [36], we examined whether SphK might be involved also in this TGFβ-induced effect. Indeed, incubation of cells with the specific SphK inhibitor SKI-2 (10 µmol/L) 45 min before TGFβ challenge, significantly reduced the increase of TPM1 and completely inhibited that of CNN1 at mRNA level (Fig. 4A). Consistently, at protein level, the blockade of SphK activity markedly reduced the increase of CNN1 and TPM1 (Fig. 4B). Analogously, in these experimental conditions, the cytokine-increased expression of other SM markers, such as TAGLN and laminin was strongly impaired and abolished, respectively (Fig. 4B). In agreement, confocal immunofluorescence analysis revealed that the significant increase of TPM1 and TAGLN expression following treatment with 0.11 µmol/L TGFβ was completely inhibited by pre-incubation with 10 µmol/L SKI-2 (Fig. 4C). In order to identify which of the two SphK isoforms is required for the TGFβ-induced effect, SphK1 and SphK2 were specifically down-regulated by RNA interference in human mesoangioblasts before being challenged for 48 h with 0.11 µmol/L TGFβ. Down-regulation of SphK1 or SphK2 resulted in a significant reduction of the expression of the SM marker CNN1 increased by the cytokine (Fig. 4D), suggesting the involvement of both isoforms in the observed effect.

Finally, in order to clarify whether the activation of SphK/S1P axis by TGFβ results in the release of the sphingolipid into the medium, we pre-treated mesoangioblasts with 15 µmol/L MK517, an inhibitor of ABCC1 transporter [15], 45 min before challenge with the cytokine. The blockade of ABCC1 transporter completely inhibited the increase of the SM marker TPM1 induced by the cytokine, identifying the transporter as responsible for this autocrine/paracrine pathway in these cells (Fig. 4E).

Overall, these latter results support the view that TGFβ-induced differentiation of mesoangioblasts towards SM depends on SphK/S1P axis.

**Figure 5:**
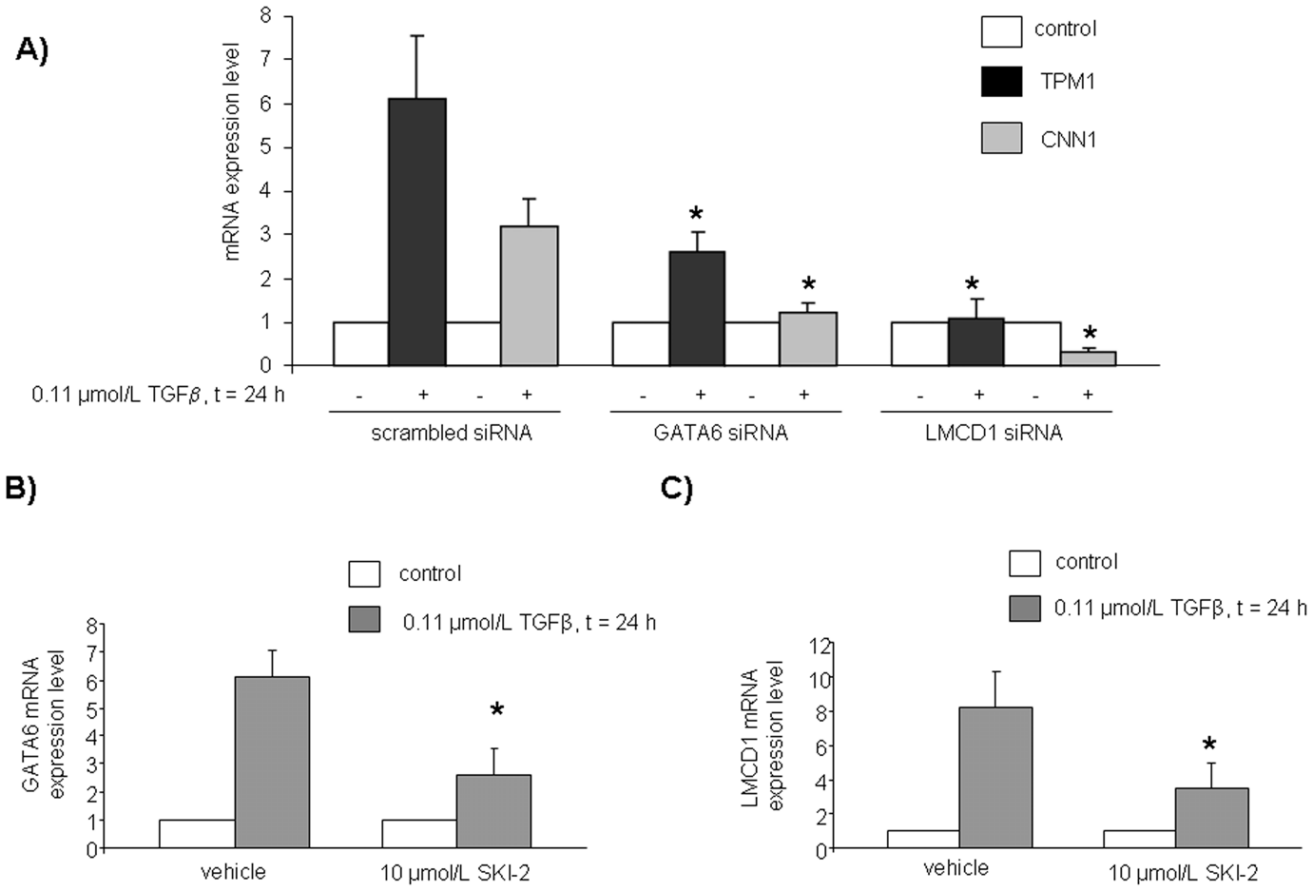
Role of GATA6 and LMCD1 transcription factors on the expression levels of SM markers induced by TGFβ in human mesoangioblasts (A) and their dependence on SphK activity (B,C). Cells transfected with scrambled-, GATA6-, LMCD1-siRNA A) and pre-treated with SKI-2 for 45 min (B,C) were stimulated with TGFβ and analyzed by Real-Time PCR.

In view of the here reported involvement of GATA6 and LMCD1 in the differentiating action of S1P, we next determined whether they were also involved in the mechanism by which TGFβ drives differentiation towards SM. As illustrated in Fig. 5A, GATA6 knock-down by specific RNA silencing strongly reduced the TGFβ-dependent enhancement of TPM1 mRNA and completely blocked that of the other key myogenic marker CNN1. Similarly, the down-regulation of LMCD1 mRNA abolished the TGFβ-induced increase of mRNA encoding for both SM markers TPM1 and CNN1. Interestingly, data presented in Fig. 5B show that TGFβ, similarly to S1P, was responsible for an increase of GATA6 mRNA content which was strongly reduced when SphK was pharmacologically inhibited. Likewise, SphK was involved in the increase of LMCD1 mRNA elicited by TGFβ (Fig. 5C). Thus, these results highlight a role for GATA6 and LMCD1 in the mechanism by which TGFβ exerts its pro-myogenic action.

**Figure 6:**
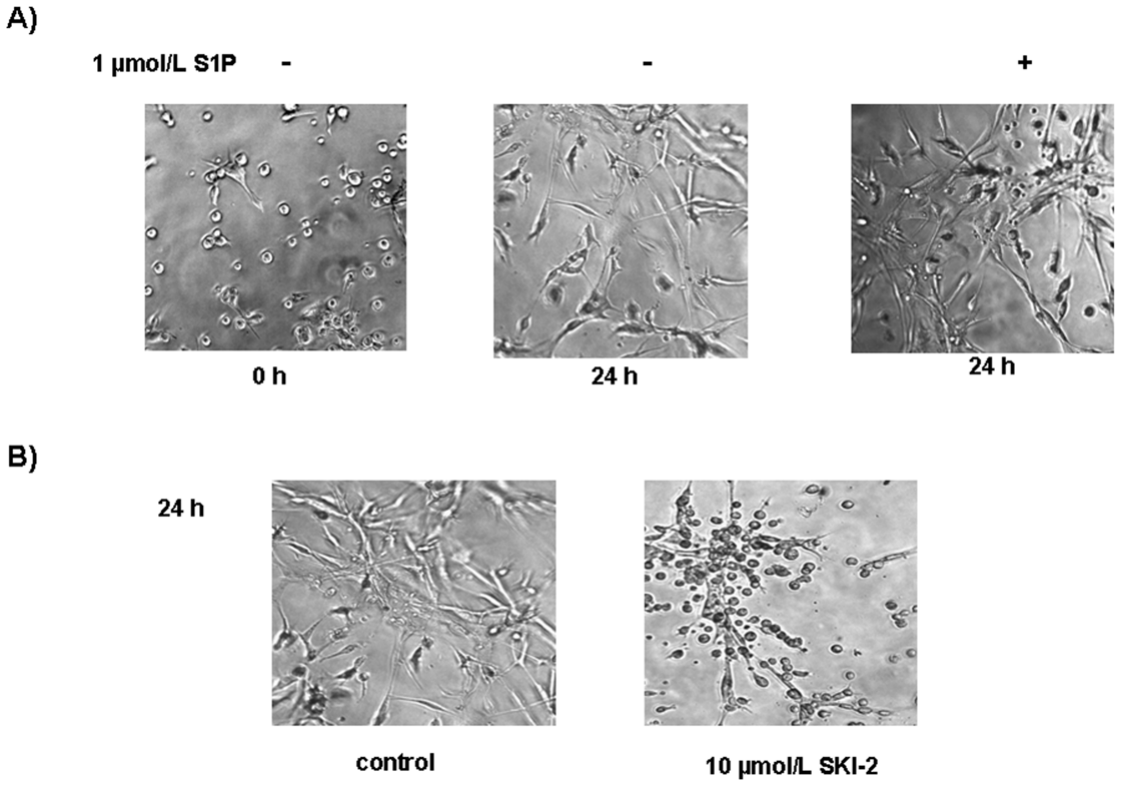
Role of SphK/S1P axis on morphogenesis of mesoangioblasts seeded on Matrigel. 20×10^3^ cells were plated in Matrigel in the presence of S1P (A) with or without SKI-2 (B) and photographed (10× magnification) at time 0 and after 24 h from plating.

Finally, we sought to evaluate the effect of S1P on morphological differentiation of mesoangioblasts. To this end, to better explore the phenotypic changes promoted by S1P in human mesoangioblasts, cells seeded in Matrigel and treated with 1 µmol/L S1P were observed at 24 h (Fig. 6A). The addition of the bioactive sphingolipid induced more profound morphological changes compared to unchallenged cells, demonstrating that, in addition to modulate the expression of SM markers, S1P acts as a real differentiating cue in these cells. Since a certain level of differentiation was also appreciable in unchallenged mesoangioblasts at 24 h, we sought to evaluate whether an endogenously produced S1P by activated SphK might be responsible for the observed effect. Indeed, incubation of the 3D matrigel culture of mesoangioblasts in the presence of 10 µmol/L SKI-2, significantly impaired the appearance of the differentiated phenotype (Fig. 6B). Although SphK plays a crucial role in the survival of mesoangioblasts [12], the presence of serum in the growing medium prevented the onset of the apoptotic cell death (data not shown). These findings confirm the crucial role of SphK/S1P axis in the induction of differentiation towards SM of these mesenchymal progenitors.

## Discussion

The interplay between extracellular signals and transcriptional regulation is a crucial nexus of control for cell lineage determination. Thus, competence to respond to the milieu of local growth factors is a fundamental determinant of the eventual fate of multipotent cells. In this regard, S1P, that can be locally produced in autocrine manner in response to the cytokine TGFβ and many diverse growth factors, deserves considerable attention in view of its pleiotropic mode of action.

Remarkably, in this study the analysis of transcriptional profile of human mesoangioblasts in response to S1P treatment not only confirms that this sphingolipid is a powerful anti-apoptotic agent but highlights for the first time its role as morphogenetic cue that, up-regulating GATA6 and LMCD1 transcription factors as well as SM contractile proteins including TPM1, CNN1, TAGLN and CALD1, triggers differentiation of these progenitor cells towards SM phenotype. In addition to the cDNA microarray study, multiple experimental evidence for the pro-differentiating activity of S1P in mesoangioblasts is here provided: the enhanced expression of transcriptional regulators and structural SM-specific proteins was validated by Real time PCR; moreover, Western blotting and confocal microscopy analysis further confirmed the S1P-mediated biological effect. Furthermore, morphological analysis of cells grown on Matrigel revealed that S1P provoked profound phenotypic modifications which were consistent with the accomplishment of a functional SM phenotype.

Interestingly, here by siRNA-mediated gene silencing, GATA6 and LMCD1 have been identified as novel transcriptional regulators of mesoangioblast differentiation towards SM. Notably, experimental evidence is provided that this novel molecular mechanism of transcriptional control of SM phenotype appearance is integral to the biological action exerted by TGFβ in these cells.

Modulation of S1P metabolism as well as S1P availability at the site of vascular lesion appears to be highly relevant for switching between different SM phenotypes in physiological and pathological conditions. Indeed, previous studies by Lockman et al. [37] have shown that treating SM cells with S1P increased their proliferation and paradoxically increased the expression of SM-specific α-actin and myosin as well as TAGLN. Subsequently, Wamhoff et al. demonstrated that S1P receptors are differentially affected by vascular injury and that S1P_1/3_ negatively regulate the phenotypical appearance of contractile protein and positively regulate proliferation, while, S1P_2_ opposes these actions [38]. Here, on the contrary, by pharmacological inhibition, S1P_2_ and S1P_1/3_ were found to be involved in the differentiating action of S1P towards SM of these progenitor mesodermal cells, in agreement with what previously reported in adipose tissue-derived mesenchymal stem cells where S1P_2_ and, at a lesser extent S1P_3_, turned out to be critical for the pro-differentiating effect of S1P [20]. The role of S1P_2_ in SM differentiation was also recently confirmed since its activation was required for the expression of SM-specific α-actin after arterial injury [39] and LARG-mediated RhoA activation leading to SM differentiation [40]. Additionally, the expression of SM-specific α-actin induced by TGFβ in C2C12 myoblasts was found to be downstream of S1P/S1P_3_ axis [41].

Moreover, S1P induces Ca^2+^-dependent contraction, proliferation and migration of SM cells [9]. Finally, genetically null mice for S1P_1_ are embryonically lethal due to impaired homing of SM cells to developing blood vessels, highlighting the importance of the S1P signalling via S1P_1_ for vascular SM cell migration [42]. In the last few years, studies examining the role of S1P on vascular function support the notion that the multiple effects elicited by this sphingolipid could represent potential therapeutic targets for angiogenesis, atherosclerosis and vascular injury. This is not only due to its multiple effects on cardio-vascular system but also because S1P plays an important role in the regulation of embryonic and adult stem cells [43]. In this regard S1P appears to play a dual role, since it maintains human embryonic stem cells in undifferentiated state [10] and acts as a differentiating cue in toti- or multi-potent cells: it induces cardiac differentiation of embryoid bodies derived from mouse embryonic stem cells [44] and up-regulates the expression of SM marker proteins of adipose tissue-derived mesenchymal stem cells [20], reinforcing the notion of a pleiotropic action of this lipid. Despite this conspicuous amount of information, little is known about the eventual transcription regulators that orchestrate the expression of tissue-specific proteins under the control of S1P, with the notable exception of cofactors of the myocardin family found implicated in differentiation of vascular rat SM cells [37]. The present finding that knock-down of GATA6 abrogates the S1P-induced expression of SM-specific proteins identifies this transcription factor as main responsible for differentiation of mesoangioblasts towards SM cells. Intriguingly, GATA6 was recently found involved in the differentiation of mesoangioblasts isolated from ventricular vessel towards cardiomyocytes [45]; interestingly, here, from cDNA microarray study troponin C mRNA was not affected (Supplemental table S1,S2), nor its protein content examined by Western blot analysis was changed (data not shown).

Additionally, in the present study in agreement with a key role of GATA6, TRPS1 which is a potent repressor of GATA transcriptional activity [29] was found dramatically down-regulated by S1P treatment. Moreover, besides GATA6, S1P significantly enhanced mRNA content of a co-regulator of GATA family protein, LMCD1, in accordance with its tissue expression pattern similar to that of GATA6 [30]. Interestingly, here GATA6 and LMCD1 were found both required for the expression of SM-specific proteins and subjected to mutual regulation of their mRNAs. Since LMCD1 has been reported to act also as a co-repressor of GATA6 [30], the present data exclude that this possibility holds true in the mechanism by which S1P promotes SM differentiation of mesoangioblasts, rather supporting its positive role on GATA6 expression.

Previously it has been shown that mesoangioblasts express many members of the TGFβ/BMP pathway and promptly differentiate into SM cells in response to TGFβ [36]. Although serum response cofactor myocardin, MRTFA and Mrf2-α and -β are known to play a fundamental role in SM differentiation [2], TGFβ-mediated differentiation of these cells into SM is myocardin-independent [3]. Two transcription factors, Necdin and Msx2, are up-regulated in response to TGFβ and are sufficient to induce some SM markers such as SM-specific myosin [3]. From the analysis of the present transcriptional profile, Necdin and Msx2 appear to be not influenced by S1P treatment, which instead was discovered to rely on GATA6 up-regulation for transmitting its biological effect, thus supporting the notion that the complex process of mesoangioblast differentiation into SM is orchestrated by multiple transcription factors.

In light of the functional cross-talk that occurs between TGFβ and S1P signaling pathways in various cell types [32]–[35], mesoangioblasts included [12], here the mechanism by which TGFβ drives mesoangioblasts towards SM phenotype was further explored. Similarly to what observed for S1P, TGFβ was found not only to enhance the expression of GATA6 and LMCD1, but also to depend on these transcriptional regulators for the up-regulation of the SM-specific proteins, TPM1 and CNN1. Importantly, the effect of TGFβ on GATA6 and LMCD1 was severely impaired when the biosynthesis of S1P was pharmacologically and genetically prevented. Hence, the here identified S1P-dependent novel molecular mechanism for triggering differentiation of mesoangioblasts towards SM cells has physiological relevance since it is exploited by TGFβ for exerting its morphogenetic action. Moreover, by specific silencing of SphK1 and SphK2, in human mesoangioblasts the pro-differentiating action of the cytokine was found to depend on both isoforms of SphKs, and the subsequent release of S1P into the media through the ABCC1 transporter as here demonstrated by pre-treatment with the inhibitor MK571 and as already reported in other cell types [15]. However, attempts made to measure S1P into the medium were unsuccessful possibly due to the partition of this sphingolipid into plasma membrane microdomains rather than its release in the extracellular environment (data not shown). Intriguingly, CNN1, here identified downstream of GATA6, is not a downstream target of Necdin or Msx2 in mesoangioblasts challenged with TGFβ 3, reinforcing the hypothesis that up-regulation of GATA6 by TGFβ is necessary to induce its full biological effect in these cells. It will be interesting to examine in a future study the eventual interplay among Necdin, Msx2 and GATA6 in the regulation of SM phenotype by TGFβ in these cells and their hierarchical relationship, possibly extending the investigation to Notch signaling, recently reported to be involved in TGFβ-dependent SM differentiation of mesenchymal stem cells [46].

Overall this study increases our knowledge of the role of S1P in these progenitors cells as differentiating cue. Intriguingly, S1P and TGFβ, here demonstrated to share molecular mechanisms essential to drive mesoangioblast differentiation, were previously identified as anti-apoptotic cues in these cells [11], [12]; moreover, SphK1 up-regulation by TGFβ was found critical for the protective role of the cytokine [12]. Whether the prevention of apoptotic death is a determining factor for the successful differentiation of these progenitors cells remains however to be established.

As regards the possible intervention in regenerative medicine, in the last few years studies examining the role of S1P on vascular function support the notion that the multiple effects elicited by this sphingolipid could represent potential therapeutic targets for angiogenesis, atherosclerosis and vascular injury. Blood vessel replacement is frequently necessary in the treatment of advanced atherosclerosis and vascular trauma. The emerging field of tissue engineering represents a mean to construct functional grafts when autologous tissue is unavailable due to donor site morbidity. Replacement vessels are engineered in vitro by seeding polymeric tubular scaffolds with autologous endothelial and SM cells [47]. The results of the present study suggest that mesoangioblasts, relatively easy to obtain with an extensive in vitro proliferation capacity, could be potentially used as a source of SM cells for cellular therapeutics and tissue engineering.

In conclusion, in this study we demonstrate that S1P induces differentiation of mesoangioblasts mesenchymal stem cells towards a SM phenotype. Importantly, GATA6 up-regulation, together with that of LMCD1, was identified as a novel mechanism of transcriptional regulation that drives SM differentiation of mesoangioblasts. Moreover, we provide evidence that GATA6 plays also a role in the mechanism by which TGFβ trigger SM differentiation of mesoangioblasts and that S1P biosynthesis is required for the accomplishment of the biological action of the cytokine. The present results represent an original contribution to the knowledge of the molecular mechanisms that regulate differentiation of these progenitors cells and disclose SphK/S1P axis as an attractive novel target to stimulate differentiation of mesoangioblasts towards a SM contractile phenotype.

## Supporting Information

Table S1 Genes up-regulated by S1P treatment in human mesoangioblasts
(DOC)

Table S2 Genes down-regulated by S1P treatment in human mesoangioblasts
(DOC)
