# Supplementary material for: Sphingosine 1-Phosphate Induces Differentiation of Mesoangioblasts towards Smooth Muscle. A Role for GATA6
Source: PLoS One. 2011 May 24;6(5):e20389. doi: 10.1371/journal.pone.0020389 (PMC3101247; doi:10.1371/journal.pone.0020389)
Supplement: Table S1 — Genes up-regulated by S1P treatment in human mesoangioblasts (DOC) [file pone.0020389.s001.doc]

**"SUPPLEMENTAL MATERIAL."**

**Table S1** Genes up-regulated by S1P treatment in human mesoangioblasts

GEO accession number GSE17596
 <http://www.ncbi.nlm.nih.gov/geo/query/acc.cgi?token=tpivteiccuwkspi&acc=GSE17596>

| **Gene description** | **AgilentID** | **GeneNames** | **d-value** |
| --- | --- | --- | --- |
| solute carrier family 38, member 1 (SLC38A1), transcript variant 1, mRNA [NM_030674] | A_24_P261734 | SLC38A1 | 7.92 |
| tropomyosin 1 (alpha) (TPM1), transcript variant 3, mRNA [NM_001018004] | A_32_P89709 | TPM1 | 7.66 |
| tropomyosin 1 (alpha) (TPM1), transcript variant 3, mRNA [NM_001018004] | A_23_P391586 | TPM1 | 7.29 |
| Unknown | A_23_P123234 | Unknown | 7.05 |
| GRAM domain containing 3 (GRAMD3), mRNA [NM_023927] | A_23_P22350 | GRAMD3 | 6.99 |
| Unknown | A_23_P123234 | Unknown | 6.88 |
| Actin-like protein (Fragment). [Source:Uniprot/SPTREMBL;Acc:Q562T7] [ENST00000375923] | A_24_P410017 | ENST00000375923 | 6.69 |
| tumor necrosis factor receptor superfamily, member 11b (osteoprotegerin) (TNFRSF11B), mRNA [NM_002546] | A_24_P192485 | TNFRSF11B | 6.61 |
| keratin associated protein 2-4, mRNA (cDNA clone MGC:74790 IMAGE:3907481), complete cds. [BC063625] | A_32_P24376 | KRTAP2-4 | 6.51 |
| collagen, type XI, alpha 1 (COL11A1), transcript variant B, mRNA [NM_080629] | A_23_P11806 | COL11A1 | 6.45 |
| brain expressed X-linked 2 (BEX2), mRNA [NM_032621] | A_23_P22735 | BEX2 | 6.40 |
| PRKC, apoptosis, WT1, regulator (PAWR), mRNA [NM_002583] | A_23_P53663 | PAWR | 6.39 |
| Unknown | A_23_P123234 | Unknown | 6.35 |
| UDP-Gal:betaGlcNAc beta 1,4- galactosyltransferase, polypeptide 4 (B4GALT4), transcript variant 1, mRNA [NM_212543] | A_32_P103945 | B4GALT4 | 6.34 |
| collagen, type IV, alpha 2 (COL4A2), mRNA [NM_001846] | A_23_P205031 | COL4A2 | 6.20 |
| Ras association (RalGDS/AF-6) domain family 7 (RASSF7), mRNA [NM_003475] | A_23_P52589 | RASSF7 | 6.20 |
| mitogen-activated protein kinase kinase kinase 14 (MAP3K14), mRNA [NM_003954] | A_23_P207319 | MAP3K14 | 6.16 |
| Unknown | A_23_P123234 | Unknown | 6.16 |
| Tissue factor pathway inhibitor 2 precursor (TFPI-2) (Placental protein 5) (PP5). [Source:Uniprot/SWISSPROT;Acc:P48307] [ENST00000222543] | A_24_P95070 | TFPI2 | 6.14 |
| protein phosphatase 1, regulatory (inhibitor) subunit 13 like (PPP1R13L), mRNA [NM_006663] | A_23_P119095 | PPP1R13L | 6.05 |
| calponin 1, basic, smooth muscle (CNN1), mRNA [NM_001299] | A_23_P125233 | CNN1 | 6.02 |
| metallothionein 2A (MT2A), mRNA [NM_005953] | A_24_P361896 | MT2A | 5.99 |
| Unknown | A_23_P123234 | Unknown | 5.97 |
| GATA binding protein 6 (GATA6), mRNA [NM_005257] | A_23_P304450 | GATA6 | 5.83 |
| Unknown | A_23_P123234 | Unknown | 5.77 |
| collagen, type XII, alpha 1 (COL12A1), transcript variant long, mRNA [NM_004370] | A_23_P214168 | COL12A1 | 5.75 |
| Unknown | A_23_P123234 | Unknown | 5.70 |
| lysyl oxidase (LOX), mRNA [NM_002317] | A_23_P122216 | LOX | 5.70 |
| dickkopf homolog 1 (Xenopus laevis) (DKK1), mRNA [NM_012242] | A_23_P24129 | DKK1 | 5.62 |
| sulfatase 1 (SULF1), mRNA [NM_015170] | A_23_P43164 | SULF1 | 5.60 |
| diacylglycerol kinase, delta 130kDa (DGKD), transcript variant 2, mRNA [NM_152879] | A_23_P210253 | DGKD | 5.59 |
| keratin 34 (KRT34), mRNA [NM_021013] | A_23_P101054 | KRT34 | 5.59 |
| dishevelled associated activator of morphogenesis 2 (DAAM2), mRNA [NM_015345] | A_32_P52785 | DAAM2 | 5.54 |
| ATPase, Class V, type 10A (ATP10A), mRNA [NM_024490] | A_24_P215765 | ATP10A | 5.52 |
| Src homology 2 domain containing adaptor protein B (SHB), mRNA [NM_003028] | A_24_P146575 | SHB | 5.50 |
| Unknown | A_23_P123234 | Unknown | 5.50 |
| actin-like protein (FKSG30), mRNA [NM_001017421] | A_32_P155776 | FKSG30 | 5.46 |
| KIAA1199 (KIAA1199), mRNA [NM_018689] | A_23_P324754 | KIAA1199 | 5.45 |
| PREDICTED: similar to cytoplasmic beta-actin (LOC646048), mRNA [XR_019608] | A_24_P461497 | LOC646048 | 5.44 |
| tumor necrosis factor receptor superfamily, member 11b (osteoprotegerin) (TNFRSF11B), mRNA [NM_002546] | A_23_P71530 | TNFRSF11B | 5.42 |
| tumor necrosis factor receptor superfamily, member 11b (osteoprotegerin) (TNFRSF11B), mRNA [NM_002546] | A_23_P71530 | TNFRSF11B | 5.42 |
| tropomyosin 1 (alpha) (TPM1), transcript variant 5, mRNA [NM_000366] | A_24_P44462 | TPM1 | 5.36 |
| LIM and cysteine-rich domains 1 (LMCD1), mRNA [NM_014583] | A_23_P6771 | LMCD1 | 5.34 |
| actin, beta (ACTB), mRNA [NM_001101] | A_32_P137939 | ACTB | 5.29 |
| transcription factor 15 (basic helix-loop-helix) (TCF15), mRNA [NM_004609] | A_23_P254816 | TCF15 | 5.26 |
| serum/glucocorticoid regulated kinase (SGK), mRNA [NM_005627] | A_23_P19673 | SGK | 5.21 |
| Unknown | A_23_P123234 | Unknown | 5.20 |
| mRNA; cDNA DKFZp451G189 (from clone DKFZp451G189). [AL833294] | A_23_P310094 | SYNPO2 | 5.17 |
| cDNA FLJ31353 fis, clone MESAN2000264. [AK055915] | A_24_P924697 | AK055915 | 4.96 |
| brain expressed, X-linked 1 (BEX1), mRNA [NM_018476] | A_23_P159952 | BEX1 | 4.94 |
| serpin peptidase inhibitor, clade E (nexin, plasminogen activator inhibitor type 1), member 1 (SERPINE1), mRNA [NM_000602] | A_24_P158089 | SERPINE1 | 4.94 |
| chromosome 12 open reading frame 49 (C12orf49), mRNA [NM_024738] | A_23_P13797 | C12orf49 | 4.94 |
| secreted frizzled-related protein 4 (SFRP4), mRNA [NM_003014] | A_23_P215328 | SFRP4 | 4.85 |
| neuron navigator 3 (NAV3), mRNA [NM_014903] | A_23_P13740 | NAV3 | 4.79 |
| insulin-like growth factor binding protein 7 (IGFBP7), mRNA [NM_001553] | A_23_P353035 | IGFBP7 | 4.79 |
| tumor necrosis factor receptor superfamily, member 11b (osteoprotegerin) (TNFRSF11B), mRNA [NM_002546] | A_23_P71530 | TNFRSF11B | 4.78 |
| lactate dehydrogenase A (LDHA), mRNA [NM_005566] | A_32_P231391 | LDHA | 4.78 |
| KDEL (Lys-Asp-Glu-Leu) containing 1 (KDELC1), mRNA [NM_024089] | A_23_P128613 | KDELC1 | 4.76 |
| glucuronidase, beta-like 2, mRNA (cDNA clone IMAGE:6047050). [BC065547] | A_24_P614148 | GUSBL2 | 4.73 |
| Unknown | A_23_P123234 | Unknown | 4.73 |
| protein kinase, AMP-activated, gamma 2 non-catalytic subunit (PRKAG2), transcript variant a, mRNA [NM_016203] | A_23_P314760 | PRKAG2 | 4.72 |
| secretory leukocyte peptidase inhibitor (SLPI), mRNA [NM_003064] | A_23_P91230 | SLPI | 4.71 |
| heart alpha-kinase (HAK), mRNA [NM_052947] | A_23_P15876 | ALPK2 | 4.71 |
| caldesmon 1 (CALD1), transcript variant 1, mRNA [NM_033138] | A_24_P921366 | CALD1 | 4.70 |
| tropomyosin 1 (alpha) (TPM1), transcript variant 5, mRNA [NM_000366] | A_23_P363344 | TPM1 | 4.68 |
| lysyl oxidase (LOX), mRNA [NM_002317] | A_23_P122216 | LOX | 4.67 |
| lysyl oxidase (LOX), mRNA [NM_002317] | A_23_P122216 | LOX | 4.64 |
| UI-H-BW1-anf-g-07-0-UI.s1 NCI_CGAP_Sub7 cDNA clone IMAGE:3082188 3', mRNA sequence [BF514513] | A_32_P139894 | BF514513 | 4.62 |
| kinesin family member 23 (KIF23), transcript variant 1, mRNA [NM_138555] | A_23_P48835 | KIF23 | 4.61 |
| polycystic kidney disease 2 (autosomal dominant) (PKD2), mRNA [NM_000297] | A_24_P106112 | PKD2 | 4.58 |
| KIAA1191 (KIAA1191), transcript variant 1, mRNA [NM_020444] | A_23_P383910 | KIAA1191 | 4.57 |
| sema domain, transmembrane domain (TM), and cytoplasmic domain, (semaphorin) 6D (SEMA6D), transcript variant 4, mRNA [NM_153618] | A_23_P420442 | SEMA6D | 4.55 |
| superoxide dismutase 2, mitochondrial (SOD2), nuclear gene encoding mitochondrial protein, transcript variant 2, mRNA [NM_001024465] | A_23_P134176 | SOD2 | 4.53 |
| KDEL (Lys-Asp-Glu-Leu) containing 1 (KDELC1), mRNA [NM_024089] | A_23_P128613 | KDELC1 | 4.51 |
| neuron navigator 3 (NAV3), mRNA [NM_014903] | A_24_P318160 | NAV3 | 4.51 |
| lysyl oxidase (LOX), mRNA [NM_002317] | A_23_P122216 | LOX | 4.51 |
| tropomyosin 1 (alpha) (TPM1), transcript variant 3, mRNA [NM_001018004] | A_23_P206018 | TPM1 | 4.50 |
| cDNA FLJ42879 fis, clone BRHIP3001283. [AK124869] | A_24_P686965 | SH2D5 | 4.50 |
| KIAA1199 (KIAA1199), mRNA [NM_018689] | A_32_P161855 | KIAA1199 | 4.49 |
| lysyl oxidase (LOX), mRNA [NM_002317] | A_23_P122216 | LOX | 4.48 |
| adrenomedullin (ADM), mRNA [NM_001124] | A_23_P127948 | ADM | 4.48 |
| cDNA clone MGC:102982 IMAGE:30378151, complete cds. [BC092424] | A_24_P238744 | LOC644936 | 4.48 |
| polo-like kinase 2 (Drosophila) (PLK2), mRNA [NM_006622] | A_23_P30254 | PLK2 | 4.48 |
| solute carrier family 25, member 28 (SLC25A28), mRNA [NM_031212] | A_23_P75220 | SLC25A28 | 4.48 |
| WW and C2 domain containing 2 (WWC2), mRNA [NM_024949] | A_23_P92569 | WWC2 | 4.48 |
| gremlin 1, cysteine knot superfamily, homolog (Xenopus laevis) (GREM1), mRNA [NM_013372] | A_23_P432947 | GREM1 | 4.42 |
| tissue factor pathway inhibitor 2 (TFPI2), mRNA [NM_006528] | A_23_P393620 | TFPI2 | 4.42 |
| ribosomal protein S2 (RPS2), mRNA [NM_002952] | A_23_P106708 | RPS2 | 4.39 |
| transgelin (TAGLN), transcript variant 1, mRNA [NM_001001522] | A_23_P87013 | TAGLN | 4.39 |
| lysyl oxidase (LOX), mRNA [NM_002317] | A_23_P122216 | LOX | 4.37 |
| metallothionein 2A (MT2A), mRNA [NM_005953] | A_23_P106844 | MT2A | 4.35 |
| KDEL (Lys-Asp-Glu-Leu) containing 1 (KDELC1), mRNA [NM_024089] | A_23_P128613 | KDELC1 | 4.33 |
| follistatin-like 1 (FSTL1), mRNA [NM_007085] | A_23_P212696 | FSTL1 | 4.32 |
| AGENCOURT_14122420 NIH_MGC_187 cDNA clone IMAGE:30381796 5', mRNA sequence [CD241953] | A_24_P354523 | CD241953 | 4.32 |
| ankyrin repeat domain 13A (ANKRD13A), mRNA [NM_033121] | A_24_P98975 | ANKRD13A | 4.31 |
| WD repeat domain 1 (WDR1), transcript variant 1, mRNA [NM_017491] | A_23_P213000 | WDR1 | 4.31 |
| Metallothionein-2 (MT-2) (Metallothionein-II) (MT-II) (Metallothionein-2A). [Source:Uniprot/SWISSPROT;Acc:P02795] [ENST00000245185] | A_23_P252413 | MT2A | 4.30 |
| transmembrane protein 38A (TMEM38A), mRNA [NM_024074] | A_24_P202748 | TMEM38A | 4.29 |
| lysyl oxidase (LOX), mRNA [NM_002317] | A_23_P122216 | LOX | 4.28 |
| proteasome (prosome, macropain) 26S subunit, non-ATPase, 12 (PSMD12), mRNA [NM_002816] | A_23_P77876 | PSMD12 | 4.27 |
| tumor-associated calcium signal transducer 2 (TACSTD2), mRNA [NM_002353] | A_23_P149529 | TACSTD2 | 4.27 |
| PREDICTED: similar to cytoplasmic beta-actin (LOC649003), mRNA [XR_018969] | A_24_P247175 | LOC649003 | 4.20 |
| cysteine and glycine-rich protein 2 (CSRP2), mRNA [NM_001321] | A_23_P44724 | CSRP2 | 4.18 |
| lysyl oxidase-like 3 (LOXL3), mRNA [NM_032603] | A_23_P39799 | LOXL3 | 4.17 |
| lysyl oxidase (LOX), mRNA [NM_002317] | A_23_P122216 | LOX | 4.17 |
| KDEL (Lys-Asp-Glu-Leu) containing 1 (KDELC1), mRNA [NM_024089] | A_23_P128613 | KDELC1 | 4.15 |
| Unknown | A_24_P777185 | Unknown | 4.15 |
| cDNA FLJ35556 fis, clone SPLEN2004844. [AK092875] | A_32_P194164 | AK092875 | 4.15 |
| transgelin (TAGLN), transcript variant 1, mRNA [NM_001001522] | A_23_P87011 | TAGLN | 4.13 |
| tuftelin 1 (TUFT1), mRNA [NM_020127] | A_23_P371824 | TUFT1 | 4.12 |
| dickkopf homolog 3 (Xenopus laevis) (DKK3), transcript variant 1, mRNA [NM_015881] | A_24_P261417 | DKK3 | 4.12 |
| tubulin, beta 2C (TUBB2C), mRNA [NM_006088] | A_32_P187327 | TUBB2C | 4.12 |
| zinc finger CCCH-type containing 12A (ZC3H12A), mRNA [NM_025079] | A_23_P326160 | ZC3H12A | 4.11 |
| DNA polymerase-transactivated protein 6 (DNAPTP6), mRNA [NM_015535] | A_23_P131255 | DNAPTP6 | 4.11 |
| lysyl oxidase (LOX), mRNA [NM_002317] | A_23_P122216 | LOX | 4.09 |
| asparagine synthetase (ASNS), transcript variant 2, mRNA [NM_001673] | A_23_P145694 | ASNS | 4.09 |
| dihydrofolate reductase (DHFR), mRNA [NM_000791] | A_32_P211045 | DHFR | 4.08 |
| tumor necrosis factor receptor superfamily, member 11b (osteoprotegerin) (TNFRSF11B), mRNA [NM_002546] | A_23_P71530 | TNFRSF11B | 4.08 |
| lysyl oxidase (LOX), mRNA [NM_002317] | A_23_P122216 | LOX | 4.07 |
| four and a half LIM domains 2 (FHL2), transcript variant 5, mRNA [NM_001039492] | A_23_P108751 | FHL2 | 4.07 |
| KDEL (Lys-Asp-Glu-Leu) containing 1 (KDELC1), mRNA [NM_024089] | A_23_P128613 | KDELC1 | 4.07 |
| cDNA FLJ39878 fis, clone SPLEN2016045, moderately similar to mRNA for Hrs. [AK097197] | A_24_P937319 | HGS | 4.06 |
| LIM and senescent cell antigen-like domains 2 (LIMS2), mRNA [NM_017980] | A_23_P142796 | LIMS2 | 4.05 |
| vang-like 1 (van gogh, Drosophila) (VANGL1), mRNA [NM_138959] | A_23_P103795 | VANGL1 | 4.03 |
| Fas (TNF receptor superfamily, member 6) (FAS), transcript variant 1, mRNA [NM_000043] | A_23_P63896 | FAS | 4.02 |
| Tetratricopeptide repeat protein KIAA0372 (TPR repeat protein KIAA0372). [Source:Uniprot/SWISSPROT;Acc:Q6PGP7] [ENST00000380021] | A_23_P61854 | KIAA0372 | 4.02 |
| tumor protein D52-like 1 (TPD52L1), transcript variant 2, mRNA [NM_001003395] | A_23_P31143 | TPD52L1 | 4.01 |
| formin homology 2 domain containing 3 (FHOD3), mRNA [NM_025135] | A_32_P34444 | FHOD3 | 4.00 |
| Novel protein (Hypothetical protein RP13-15M17.2). [Source:Uniprot/SPTREMBL;Acc:Q5SNT3] [ENST00000340381] | A_24_P522631 | ENST00000340381 | 4.00 |
| PREDICTED: similar to T04C12.5 (LOC441783), mRNA [XR_018872] | A_24_P187626 | LOC441783 | 4.00 |
| mesoderm specific transcript homolog (mouse) (MEST), transcript variant 1, mRNA [NM_002402] | A_23_P156970 | MEST | 3.98 |
| protein tyrosine phosphatase-like (proline instead of catalytic arginine), member A (PTPLA), mRNA [NM_014241] | A_23_P161352 | PTPLA | 3.98 |
| KDEL (Lys-Asp-Glu-Leu) containing 1 (KDELC1), mRNA [NM_024089] | A_23_P128613 | KDELC1 | 3.97 |
| ubiquitin C (UBC), mRNA [NM_021009] | A_24_P681301 | UBC | 3.96 |
| calcium channel, voltage-dependent, L type, alpha 1C subunit (CACNA1C), mRNA [NM_000719] | A_23_P373028 | CACNA1C | 3.96 |
| full-length cDNA clone CS0DN005YK16 of Adult brain of (human). [CR613654] | A_32_P840463 | CR613654 | 3.95 |
| H.sapiens a-myb mRNA. [X66087] | A_23_P43157 | MYBL1 | 3.95 |
| RNA binding motif protein 14 (RBM14), mRNA [NM_006328] | A_23_P150255 | RBM14 | 3.95 |
| neuropilin 2 (NRP2), transcript variant 1, mRNA [NM_201266] | A_23_P209669 | NRP2 | 3.94 |
| Homo sapiens, clone IMAGE:4052494, mRNA. [BC006998] | A_24_P179336 | BC006998 | 3.94 |
| ribosomal protein L31 (RPL31), mRNA [NM_000993] | A_24_P213783 | RPL31 | 3.93 |
| optineurin (OPTN), transcript variant 1, mRNA [NM_001008211] | A_23_P1461 | OPTN | 3.93 |
| cingulin-like 1 (CGNL1), mRNA [NM_032866] | A_23_P163306 | CGNL1 | 3.93 |
| transforming, acidic coiled-coil containing protein 1 (TACC1), mRNA [NM_006283] | A_24_P98249 | TACC1 | 3.92 |
| leiomodin 1 (smooth muscle) (LMOD1), mRNA [NM_012134] | A_23_P201940 | LMOD1 | 3.92 |
| PREDICTED: similar to 60S ribosomal protein L26 (LOC646161), mRNA [XR_018048] | A_32_P190488 | LOC400055 | 3.91 |
| cytokine induced apoptosis inhibitor 1 (CIAPIN1), mRNA [NM_020313] | A_32_P204381 | CIAPIN1 | 3.87 |
| hect domain and RLD 4 (HERC4), transcript variant 3, mRNA [NM_001017972] | A_24_P346807 | HERC4 | 3.87 |
| polymerase I and transcript release factor (PTRF), mRNA [NM_012232] | A_23_P394064 | PTRF | 3.87 |
| ATPase, H+ transporting, lysosomal 31kDa, V1 subunit E1 (ATP6V1E1), transcript variant 1, mRNA [NM_001696] | A_23_P143551 | ATP6V1E1 | 3.86 |
| natriuretic peptide receptor C/guanylate cyclase C (atrionatriuretic peptide receptor C) (NPR3), mRNA [NM_000908] | A_23_P253536 | NPR3 | 3.86 |
| connective tissue growth factor (CTGF), mRNA [NM_001901] | A_23_P19663 | CTGF | 3.85 |
| chromosome 10 open reading frame 11 (C10orf11), mRNA [NM_032024] | A_23_P113034 | C10orf11 | 3.85 |
| KDEL (Lys-Asp-Glu-Leu) containing 1 (KDELC1), mRNA [NM_024089] | A_23_P128613 | KDELC1 | 3.85 |
| ornithine decarboxylase antizyme 1 (OAZ1), mRNA [NM_004152] | A_24_P918808 | OAZ1 | 3.85 |
| apoptosis related protein APR-4 mRNA, partial cds. [AF144054] | A_24_P221914 | AF144054 | 3.83 |
| integrin, alpha 11 (ITGA11), transcript variant 1, mRNA [NM_001004439] | A_23_P206022 | ITGA11 | 3.83 |
| CCR4-NOT transcription complex, subunit 7 (CNOT7), transcript variant 1, mRNA [NM_013354] | A_23_P394166 | CNOT7 | 3.81 |
| PWP2 periodic tryptophan protein homolog (yeast) (PWP2), mRNA [NM_005049] | A_23_P102925 | PWP2 | 3.81 |
| cDNA clone IMAGE:6141118, partial cds. [BC053363] | A_23_P78244 | BC053363 | 3.80 |
| fibroblast growth factor 1 (acidic) (FGF1), transcript variant 1, mRNA [NM_000800] | A_24_P111106 | FGF1 | 3.80 |
| pleckstrin homology domain containing, family G (with RhoGef domain) member 3 (PLEKHG3), mRNA [NM_015549] | A_23_P76901 | PLEKHG3 | 3.80 |
| protein tyrosine phosphatase, receptor type, f polypeptide (PTPRF), interacting protein (liprin), alpha 1 (PPFIA1), transcript variant 2, mRNA [NM_003626] | A_23_P75509 | PPFIA1 | 3.79 |
| Unknown | A_24_P384200 | Unknown | 3.79 |
| jerky homolog (mouse) (JRK), transcript variant 1, mRNA [NM_003724] | A_23_P334635 | JRK | 3.79 |
| chromosome 5 open reading frame 23 (C5orf23), mRNA [NM_024563] | A_23_P58676 | C5orf23 | 3.79 |
| integrin, alpha V (vitronectin receptor, alpha polypeptide, antigen CD51) (ITGAV), mRNA [NM_002210] | A_23_P50907 | ITGAV | 3.79 |
| coiled-coil domain containing 99 (CCDC99), mRNA [NM_017785] | A_23_P41948 | CCDC99 | 3.78 |
| major histocompatibility complex, class I, C, mRNA (cDNA clone MGC:2285 IMAGE:3345005), complete cds. [BC002463] | A_23_P113716 | HLA-C | 3.78 |
| ATPase family, AAA domain containing 2 (ATAD2), mRNA [NM_014109] | A_23_P216068 | ATAD2 | 3.78 |
| cyclin-dependent kinase 7 (MO15 homolog, Xenopus laevis, cdk-activating kinase) (CDK7), mRNA [NM_001799] | A_23_P133585 | CDK7 | 3.76 |
| tripartite motif-containing 37 (TRIM37), transcript variant 1, mRNA [NM_015294] | A_23_P21230 | TRIM37 | 3.76 |
| mortality factor 4 like 2 (MORF4L2), mRNA [NM_012286] | A_23_P114405 | MORF4L2 | 3.76 |
| Kruppel-like factor 5 (intestinal) (KLF5), mRNA [NM_001730] | A_23_P53891 | KLF5 | 3.76 |
| Rho GTPase activating protein 17 (ARHGAP17), transcript variant 1, mRNA [NM_001006634] | A_23_P15113 | ARHGAP17 | 3.75 |
| tripartite motif-containing 35 (TRIM35), transcript variant 2, mRNA [NM_171982] | A_23_P502553 | TRIM35 | 3.74 |
| glycogenin 1 (GYG1), mRNA [NM_004130] | A_23_P384517 | GYG1 | 3.73 |
| caldesmon 1 (CALD1), transcript variant 1, mRNA [NM_033138] | A_23_P42575 | CALD1 | 3.73 |
| nexilin (F actin binding protein) (NEXN), mRNA [NM_144573] | A_24_P409971 | NEXN | 3.72 |
| signal transducer and activator of transcription 1, 91kDa (STAT1), transcript variant beta, mRNA [NM_139266] | A_24_P274270 | STAT1 | 3.71 |
| solute carrier family 4 (anion exchanger), member 1, adaptor protein (SLC4A1AP), mRNA [NM_018158] | A_23_P56810 | SLC4A1AP | 3.69 |
| EGF-containing fibulin-like extracellular matrix protein 1 (EFEMP1), transcript variant 1, mRNA [NM_004105] | A_23_P501007 | EFEMP1 | 3.68 |
| keratin associated protein 1-5 (KRTAP1-5), mRNA [NM_031957] | A_23_P118842 | KRTAP1-5 | 3.68 |
| SAR1 gene homolog B (S. cerevisiae) (SAR1B), transcript variant 1, mRNA [NM_001033503] | A_23_P92842 | SAR1B | 3.66 |
| RAB guanine nucleotide exchange factor (GEF) 1 (RABGEF1), mRNA [NM_014504] | A_24_P232049 | RABGEF1 | 3.66 |
| capping protein (actin filament) muscle Z-line, alpha 1 (CAPZA1), mRNA [NM_006135] | A_24_P310894 | CAPZA1 | 3.66 |
| tropomyosin 3 (TPM3), transcript variant 3, mRNA [NM_001043352] | A_32_P29784 | TPM3 | 3.66 |
| tumor necrosis factor receptor superfamily, member 11b (osteoprotegerin) (TNFRSF11B), mRNA [NM_002546] | A_23_P71530 | TNFRSF11B | 3.64 |
| parvin, alpha (PARVA), mRNA [NM_018222] | A_23_P47642 | PARVA | 3.64 |
| SUMO1/sentrin/SMT3 specific peptidase 3 (SENP3), mRNA [NM_015670] | A_23_P164000 | SENP3 | 3.64 |
| Unknown | A_32_P179910 | THC2667666 | 3.63 |
| cDNA clone IMAGE:5271968. [BC092511] | A_24_P922631 | LOC133874 | 3.63 |
| frizzled homolog 7 (Drosophila) (FZD7), mRNA [NM_003507] | A_23_P209449 | FZD7 | 3.63 |
| sorting nexin 25 (SNX25), mRNA [NM_031953] | A_24_P303097 | SNX25 | 3.62 |
| actin, gamma 1 (ACTG1), mRNA [NM_001614] | A_32_P175198 | ACTG1 | 3.62 |
| furry homolog (Drosophila) (FRY), mRNA [NM_023037] | A_23_P105862 | FRY | 3.60 |
| laminin, gamma 1 (formerly LAMB2) (LAMC1), mRNA [NM_002293] | A_23_P201628 | LAMC1 | 3.60 |
| late cornified envelope 1A (LCE1A), mRNA [NM_178348] | A_23_P404685 | LCE1A | 3.60 |
| tubulin, beta 6 (TUBB6), mRNA [NM_032525] | A_23_P254271 | TUBB6 | 3.59 |
| Unknown | A_32_P118896 | Unknown | 3.59 |
| lectin, galactoside-binding, soluble, 8 (galectin 8) (LGALS8), transcript variant 1, mRNA [NM_006499] | A_23_P63026 | LGALS8 | 3.59 |
| Unknown | A_24_P161393 | Unknown | 3.58 |
| PAK1 interacting protein 1 (PAK1IP1), mRNA [NM_017906] | A_23_P122674 | PAK1IP1 | 3.58 |
| membrane associated guanylate kinase, WW and PDZ domain containing 2 (MAGI2), mRNA [NM_012301] | A_23_P21376 | MAGI2 | 3.57 |
| transmembrane protein 150 (TMEM150), transcript variant 1, mRNA [NM_001031738] | A_24_P291401 | TMEM150 | 3.57 |
| nucleoporin 62kDa (NUP62), transcript variant 1, mRNA [NM_153719] | A_24_P322444 | NUP62 | 3.57 |
| Plasminogen activator inhibitor 2 precursor (PAI-2) (Placental plasminogen activator inhibitor) (Monocyte Arg-serpin) (Urokinase inhibitor). [Source:Uniprot/SWISSPROT;Acc:P05120] [ENST00000299502] | A_23_P153185 | SERPINB2 | 3.56 |
| oxytocin receptor (OXTR), mRNA [NM_000916] | A_23_P132619 | OXTR | 3.55 |
| translocase of inner mitochondrial membrane 23 homolog (yeast) (TIMM23), mRNA [NM_006327] | A_23_P373649 | TIMM23 | 3.55 |
| actin, alpha 2, smooth muscle, aorta (ACTA2), mRNA [NM_001613] | A_23_P150053 | ACTA2 | 3.55 |
| chromosome 5 open reading frame 30 (C5orf30), mRNA [NM_033211] | A_23_P122007 | C5orf30 | 3.53 |
| cysteine rich transmembrane BMP regulator 1 (chordin-like) (CRIM1), mRNA [NM_016441] | A_23_P51105 | CRIM1 | 3.53 |
| family with sequence similarity 118, member B (FAM118B), mRNA [NM_024556] | A_23_P319492 | FAM118B | 3.53 |
| myosin, heavy chain 9, non-muscle (MYH9), mRNA [NM_002473] | A_24_P408424 | MYH9 | 3.52 |
| similar to RIKEN cDNA 4732495G21 gene (DKFZp686D0972), mRNA [NM_001017992] | A_24_P6903 | DKFZp686D0972 | 3.52 |
| cDNA FLJ13495 fis, clone PLACE1004425. [AK023557] | A_24_P911259 | AK023557 | 3.52 |
| ectodermal-neural cortex (with BTB-like domain) (ENC1), mRNA [NM_003633] | A_24_P69095 | ENC1 | 3.51 |
| mRNA; cDNA DKFZp313A137 (from clone DKFZp313A137). [AL833309] | A_24_P892612 | AL833309 | 3.51 |
| proteasome (prosome, macropain) 26S subunit, non-ATPase, 14 (PSMD14), mRNA [NM_005805] | A_23_P165691 | PSMD14 | 3.50 |
| DEAD (Asp-Glu-Ala-Asp) box polypeptide 24 (DDX24), mRNA [NM_020414] | A_23_P151544 | DDX24 | 3.49 |
| pim-1 oncogene (PIM1), mRNA [NM_002648] | A_23_P345118 | PIM1 | 3.49 |
| cDNA: FLJ21027 fis, clone CAE07110. [AK024680] | A_24_P84130 | AK024680 | 3.48 |
| Unknown | A_32_P139021 | Unknown | 3.48 |
| Kelch repeat and BTB domain-containing protein 9. [Source:Uniprot/SWISSPROT;Acc:Q96CT2] [ENST00000288548] | A_23_P209360 | KBTBD9 | 3.47 |
| t-complex 11 (mouse)-like 1 (TCP11L1), mRNA [NM_018393] | A_23_P47247 | TCP11L1 | 3.47 |
| exosome component 9 (EXOSC9), transcript variant 2, mRNA [NM_005033] | A_23_P81121 | EXOSC9 | 3.47 |
| mucosa associated lymphoid tissue lymphoma translocation gene 1 (MALT1), transcript variant 1, mRNA [NM_006785] | A_23_P96008 | MALT1 | 3.46 |
| RNA binding motif protein 28 (RBM28), mRNA [NM_018077] | A_23_P168629 | RBM28 | 3.46 |
| ankyrin repeat domain 54 (ANKRD54), mRNA [NM_138797] | A_23_P120921 | ANKRD54 | 3.46 |
| tumor necrosis factor receptor superfamily, member 11b (osteoprotegerin) (TNFRSF11B), mRNA [NM_002546] | A_23_P71530 | TNFRSF11B | 3.46 |
| protein tyrosine phosphatase, non-receptor type 1 (PTPN1), mRNA [NM_002827] | A_23_P338890 | PTPN1 | 3.45 |
| large subunit GTPase 1 homolog (S. cerevisiae) (LSG1), mRNA [NM_018385] | A_23_P132417 | LSG1 | 3.45 |
| Ras-related protein Rab-3B. [Source:Uniprot/SWISSPROT;Acc:P20337] [ENST00000371655] | A_24_P933319 | RAB3B | 3.44 |
| LIM domain 7 (LMO7), mRNA [NM_005358] | A_24_P301146 | LMO7 | 3.43 |
| glutaminase (GLS), mRNA [NM_014905] | A_24_P294233 | GLS | 3.43 |
| PAK1 interacting protein 1 (PAK1IP1), mRNA [NM_017906] | A_23_P122674 | PAK1IP1 | 3.42 |
| leprecan-like 1 (LEPREL1), mRNA [NM_018192] | A_23_P69179 | LEPREL1 | 3.41 |
| Nedd4 family interacting protein 2 (NDFIP2), mRNA [NM_019080] | A_24_P264909 | NDFIP2 | 3.41 |
| damage-regulated autophagy modulator (DRAM), mRNA [NM_018370] | A_23_P99163 | DRAM | 3.41 |
| actin pseudogene (LOC148709) on chromosome 1 [NR_002929] | A_24_P84880 | LOC148709 | 3.40 |
| chromosome 9 open reading frame 3 (C9orf3), mRNA [NM_032823] | A_23_P73012 | C9orf3 | 3.39 |
| interferon regulatory factor 2 binding protein 1 (IRF2BP1), mRNA [NM_015649] | A_23_P90211 | IRF2BP1 | 3.38 |
| catenin (cadherin-associated protein), delta 1 (CTNND1), mRNA [NM_001331] | A_23_P251316 | CTNND1 | 3.38 |
| proteasome (prosome, macropain) activator subunit 3 (PA28 gamma; Ki) (PSME3), transcript variant 2, mRNA [NM_176863] | A_23_P164141 | PSME3 | 3.38 |
| caspase 3, apoptosis-related cysteine peptidase (CASP3), transcript variant alpha, mRNA [NM_004346] | A_23_P92410 | CASP3 | 3.37 |
| holocytochrome c synthase (cytochrome c heme-lyase) (HCCS), mRNA [NM_005333] | A_23_P257945 | HCCS | 3.37 |
| MCM4 minichromosome maintenance deficient 4 (S. cerevisiae) (MCM4), transcript variant 1, mRNA [NM_005914] | A_23_P370989 | MCM4 | 3.36 |
| protein phosphatase 2 (formerly 2A), catalytic subunit, alpha isoform (PPP2CA), mRNA [NM_002715] | A_23_P122041 | PPP2CA | 3.36 |
| triple functional domain (PTPRF interacting) (TRIO), mRNA [NM_007118] | A_24_P42603 | TRIO | 3.35 |
| four and a half LIM domains 1 (FHL1), mRNA [NM_001449] | A_23_P217326 | FHL1 | 3.34 |
| mRNA; cDNA DKFZp434C131 (from clone DKFZp434C131). [AL117482] | A_23_P206103 | ULK3 | 3.33 |
| zinc finger protein 274 (ZNF274), transcript variant ZNF274c, mRNA [NM_133502] | A_23_P153251 | ZNF274 | 3.32 |
| LIM domain 7 (LMO7), mRNA [NM_005358] | A_23_P205159 | LMO7 | 3.32 |
| ankyrin repeat and SOCS box-containing 1 (ASB1), mRNA [NM_001040445] | A_23_P165360 | ASB1 | 3.32 |
| Unknown | A_24_P725365 | Unknown | 3.32 |
| small nuclear ribonucleoprotein polypeptide A' (SNRPA1), mRNA [NM_003090] | A_32_P28685 | SNRPA1 | 3.32 |
| discs, large (Drosophila) homolog-associated protein 4 (DLGAP4), transcript variant 1, mRNA [NM_014902] | A_23_P210419 | DLGAP4 | 3.31 |
| intercellular adhesion molecule 1 (CD54), human rhinovirus receptor (ICAM1), mRNA [NM_000201] | A_23_P153320 | ICAM1 | 3.31 |
| sortilin 1 (SORT1), mRNA [NM_002959] | A_24_P325520 | SORT1 | 3.31 |
| cDNA FLJ36746 fis, clone UTERU2016757. [AK094065] | A_23_P257372 | RMND5B | 3.31 |
| cytochrome b5 reductase 3 (CYB5R3), transcript variant S, mRNA [NM_007326] | A_23_P502224 | CYB5R3 | 3.31 |
| Unknown | A_24_P475115 | Unknown | 3.31 |
| DDHD domain containing 2 [Source:RefSeq_peptide;Acc:NP_056029] [ENST00000319246] | A_23_P401238 | DDHD2 | 3.31 |
| CR740121 library (Ebert L) cDNA clone IMAGp971G1750 ; IMAGE:767753 5', mRNA sequence [CR740121] | A_32_P62796 | CR740121 | 3.31 |
| SMAD family member 3 (SMAD3), mRNA [NM_005902] | A_23_P48936 | SMAD3 | 3.30 |
| gastric inhibitory polypeptide receptor (GIPR), mRNA [NM_000164] | A_24_P373768 | GIPR | 3.30 |
| v-raf-1 murine leukemia viral oncogene homolog 1 (RAF1), mRNA [NM_002880] | A_23_P40952 | RAF1 | 3.30 |
| tubulin, gamma 1 (TUBG1), mRNA [NM_001070] | A_23_P152768 | TUBG1 | 3.29 |
| thioredoxin domain containing 14 (TXNDC14), mRNA [NM_015959] | A_24_P152635 | TXNDC14 | 3.29 |
| death inducer-obliterator 1 (DIDO1), transcript variant 1, mRNA [NM_022105] | A_23_P395426 | DIDO1 | 3.29 |
| PDZ and LIM domain 5 (PDLIM5), transcript variant 4, mRNA [NM_001011515] | A_24_P24263 | PDLIM5 | 3.28 |
| protein kinase D1 (PRKD1), mRNA [NM_002742] | A_23_P106016 | PRKD1 | 3.28 |
| solute carrier family 4, sodium bicarbonate cotransporter, member 4 (SLC4A4), mRNA [NM_003759] | A_32_P358887 | SLC4A4 | 3.28 |
| natriuretic peptide receptor C/guanylate cyclase C (atrionatriuretic peptide receptor C) (NPR3), mRNA [NM_000908] | A_23_P327451 | NPR3 | 3.27 |
| PET112-like (yeast) (PET112L), mRNA [NM_004564] | A_23_P92552 | PET112L | 3.27 |
| presenilin 1 (Alzheimer disease 3) (PSEN1), mRNA [NM_000021] | A_23_P340728 | PSEN1 | 3.26 |
| growth arrest and DNA-damage-inducible, beta (GADD45B), mRNA [NM_015675] | A_24_P239606 | GADD45B | 3.26 |
| WD repeat and HMG-box DNA binding protein 1 (WDHD1), transcript variant 1, mRNA [NM_007086] | A_23_P25873 | WDHD1 | 3.26 |
| cDNA FLJ14388 fis, clone HEMBA1002716. [AK027294] | A_24_P372189 | AK027294 | 3.26 |
| pleckstrin homology domain containing, family C (with FERM domain) member 1 (PLEKHC1), mRNA [NM_006832] | A_23_P88347 | PLEKHC1 | 3.26 |
| cysteine-rich, angiogenic inducer, 61 (CYR61), mRNA [NM_001554] | A_23_P46426 | CYR61 | 3.26 |
| Parkinson disease (autosomal recessive, early onset) 7 (PARK7), mRNA [NM_007262] | A_23_P74740 | PARK7 | 3.25 |
| v-abl Abelson murine leukemia viral oncogene homolog 2 (arg, Abelson-related gene) (ABL2), transcript variant b, mRNA [NM_007314] | A_23_P138099 | ABL2 | 3.25 |
| DAZ interacting protein 1-like (DZIP1L), mRNA [NM_173543] | A_23_P384635 | DZIP1L | 3.24 |
| full length insert cDNA clone YI48C03. [AF085848] | A_32_P128701 | AF085848 | 3.24 |
| BF960555 PM1-NN1207-071200-003-a12 NN1207 cDNA, mRNA sequence [BF960555] | A_32_P175313 | THC2521437 | 3.24 |
| Q804E3_GASAC (Q804E3) Neuronal 22 protein (Fragment), partial (18%) [THC2569153] | A_24_P398370 | THC2569153 | 3.24 |
| sphingosine-1-phosphate lyase 1 (SGPL1), mRNA [NM_003901] | A_24_P940815 | SGPL1 | 3.24 |
| TBC1 domain family, member 2 (TBC1D2), mRNA [NM_018421] | A_23_P123666 | TBC1D2 | 3.23 |
| mRNA for KIAA1372 protein, partial cds. [AB037793] | A_23_P116602 | USP35 | 3.23 |
| KIAA1632 (KIAA1632), mRNA [NM_020964] | A_24_P392146 | KIAA1632 | 3.23 |
| full-length cDNA clone CS0DF021YI14 of Fetal brain of (human). [CR620599] | A_32_P88349 | CR620599 | 3.23 |
| cycle-like factor CLIF mRNA, complete cds. [AF256215] | A_32_P399546 | ARNTL2 | 3.22 |
| KDEL (Lys-Asp-Glu-Leu) containing 1 (KDELC1), mRNA [NM_024089] | A_23_P128613 | KDELC1 | 3.22 |
| BTB and CNC homology 1, basic leucine zipper transcription factor 1 (BACH1), transcript variant 1, mRNA [NM_206866] | A_23_P211047 | BACH1 | 3.22 |
| Unknown | A_24_P255763 | Unknown | 3.22 |
| cDNA FLJ11223 fis, clone PLACE1008209. [AK002085] | A_23_P98963 | LOC144438 | 3.21 |
| collagen, type IV, alpha 1 (COL4A1), mRNA [NM_001845] | A_23_P65240 | COL4A1 | 3.21 |
| solute carrier family 38, member 1 (SLC38A1), transcript variant 1, mRNA [NM_030674] | A_23_P363399 | SLC38A1 | 3.21 |
| FAST kinase domains 2 (FASTKD2), mRNA [NM_014929] | A_23_P209649 | FASTKD2 | 3.20 |
| Rho GTPase activating protein 17 (ARHGAP17), transcript variant 1, mRNA [NM_001006634] | A_24_P401739 | ARHGAP17 | 3.20 |
| tRNA splicing endonuclease 2 homolog (S. cerevisiae) (TSEN2), mRNA [NM_025265] | A_23_P92012 | TSEN2 | 3.19 |
| transformation/transcription domain-associated protein (TRRAP), mRNA [NM_003496] | A_23_P94053 | TRRAP | 3.19 |
| solute carrier family 7 (cationic amino acid transporter, y+ system), member 1 (SLC7A1), mRNA [NM_003045] | A_24_P253251 | SLC7A1 | 3.19 |
| zinc finger, DHHC-type containing 7 (ZDHHC7), mRNA [NM_017740] | A_24_P373297 | ZDHHC7 | 3.19 |
| WD repeat domain 74 (WDR74), mRNA [NM_018093] | A_23_P36157 | WDR74 | 3.18 |
| HUMTS11 ts11 cell cycle control protein {Homo sapiens} (exp=-1; wgp=0; cg=0), partial (34%) [THC2568453] | A_32_P49284 | THC2568453 | 3.18 |
| met proto-oncogene (hepatocyte growth factor receptor) (MET), mRNA [NM_000245] | A_23_P359245 | MET | 3.18 |
| WD repeat and SOCS box-containing 2 (WSB2), mRNA [NM_018639] | A_23_P147238 | WSB2 | 3.18 |
| cDNA FLJ13694 fis, clone PLACE2000115. [AK023756] | A_24_P490911 | AK023756 | 3.17 |
| G protein-coupled receptor kinase 6 (GRK6), transcript variant 2, mRNA [NM_002082] | A_23_P257256 | GRK6 | 3.17 |
| monoglyceride lipase (MGLL), transcript variant 1, mRNA [NM_007283] | A_24_P226008 | MGLL | 3.17 |
| solute carrier family 7 (cationic amino acid transporter, y+ system), member 5 (SLC7A5), mRNA [NM_003486] | A_24_P335620 | SLC7A5 | 3.16 |
| Homo sapiens, clone IMAGE:3921709, mRNA. [BC013792] | A_23_P333802 | BC013792 | 3.16 |
| BF326020 QV1-AN0059-040800-295-e05 AN0059 cDNA, mRNA sequence [BF326020] | A_32_P85813 | BF326020 | 3.15 |
| MCM3 minichromosome maintenance deficient 3 (S. cerevisiae) associated protein (MCM3AP), mRNA [NM_003906] | A_23_P120744 | MCM3AP | 3.14 |
| zinc finger protein 26 (ZNF26), mRNA [NM_019591] | A_23_P128060 | ZNF26 | 3.13 |
| UDP-glucose ceramide glucosyltransferase (UGCG), mRNA [NM_003358] | A_23_P313389 | UGCG | 3.13 |
| filamin binding LIM protein 1 (FBLIM1), transcript variant 1, mRNA [NM_017556] | A_23_P337934 | FBLIM1 | 3.13 |
| polymerase (RNA) I polypeptide C, 30kDa (POLR1C), transcript variant 2, mRNA [NM_004875] | A_23_P70409 | POLR1C | 3.13 |
| potassium inwardly-rectifying channel, subfamily J, member 12 (KCNJ12), mRNA [NM_021012] | A_24_P339429 | KCNJ12 | 3.12 |
| hypothetical protein LOC128977 (LOC128977), mRNA [NM_173793] | A_24_P922357 | LOC128977 | 3.12 |
| acyl-Coenzyme A dehydrogenase family, member 9 (ACAD9), mRNA [NM_014049] | A_24_P191656 | ACAD9 | 3.12 |
| BG504229 602552769F1 NIH_MGC_61 cDNA clone IMAGE:4665305 5', mRNA sequence [BG504229] | A_32_P1543 | THC2522470 | 3.12 |
| F-box protein 28 (FBXO28), mRNA [NM_015176] | A_23_P137578 | FBXO28 | 3.12 |
| dynactin 5 (p25) (DCTN5), mRNA [NM_032486] | A_23_P309967 | DCTN5 | 3.11 |
| cysteine rich transmembrane BMP regulator 1 (chordin-like) (CRIM1), mRNA [NM_016441] | A_24_P11575 | CRIM1 | 3.11 |
| nucleoporin 43kDa (NUP43), transcript variant 1, mRNA [NM_198887] | A_23_P31055 | NUP43 | 3.11 |
| chromosome 9 open reading frame 5 (C9orf5), mRNA [NM_032012] | A_23_P146417 | C9orf5 | 3.11 |
| ATPase, Na+/K+ transporting, beta 1 polypeptide (ATP1B1), transcript variant 1, mRNA [NM_001677] | A_23_P62932 | ATP1B1 | 3.10 |
| nexilin (F actin binding protein) (NEXN), mRNA [NM_144573] | A_23_P200001 | NEXN | 3.10 |
| phenylalanine-tRNA synthetase-like, alpha subunit (FARSLA), mRNA [NM_004461] | A_23_P78685 | FARSLA | 3.10 |
| rhodopsin (opsin 2, rod pigment) (retinitis pigmentosa 4, autosomal dominant) (RHO), mRNA [NM_000539] | A_23_P57950 | RHO | 3.10 |
| CA428991 UI-H-FH1-bfg-h-19-0-UI.s1 NCI_CGAP_FH1 cDNA clone UI-H-FH1-bfg-h-19-0-UI 3', mRNA sequence [CA428991] | A_32_P1360 | THC2670603 | 3.10 |
| RAN, member RAS oncogene family (RAN), mRNA [NM_006325] | A_24_P47547 | RAN | 3.10 |
| serine palmitoyltransferase, long chain base subunit 2 (SPTLC2), mRNA [NM_004863] | A_23_P3146 | SPTLC2 | 3.10 |
| leucine rich repeat neuronal 3 (LRRN3), mRNA [NM_018334] | A_23_P31376 | LRRN3 | 3.10 |
| general transcription factor IIA, 2 (12kD subunit) (GTF2A2), mRNA [NM_004492] | A_24_P270525 | GTF2A2 | 3.10 |
| ceroid-lipofuscinosis, neuronal 3, juvenile (Batten, Spielmeyer-Vogt disease), mRNA (cDNA clone MGC:102840 IMAGE:5764535), complete cds. [BC111068] | A_24_P567408 | CLN3 | 3.09 |
| Unknown | A_24_P480722 | Unknown | 3.09 |
| polymerase (DNA directed), mu (POLM), mRNA [NM_013284] | A_23_P397371 | POLM | 3.09 |
| cDNA FLJ36868 fis, clone ASTRO2016681. [AK094187] | A_23_P212649 | AK094187 | 3.09 |
| damage-regulated autophagy modulator (DRAM), mRNA [NM_018370] | A_24_P355816 | DRAM | 3.08 |
| E2F transcription factor 6 (E2F6), mRNA [NM_198256] | A_32_P12610 | E2F6 | 3.08 |
| AXL receptor tyrosine kinase (AXL), transcript variant 1, mRNA [NM_021913] | A_23_P208389 | AXL | 3.07 |
| chromosome 15 open reading frame 23, mRNA (cDNA clone IMAGE:3952251), partial cds. [BC004543] | A_23_P140705 | C15orf23 | 3.07 |
| legumain (LGMN), transcript variant 2, mRNA [NM_001008530] | A_23_P25994 | LGMN | 3.07 |
| Unknown | A_32_P211253 | ENST00000331096 | 3.07 |
| cDNA FLJ38735 fis, clone KIDNE2010973, moderately similar to Mus musculus orphan transporter isoform B9 (Xtrp2) mRNA. [AK096054] | A_23_P62070 | SLC6A19 | 3.07 |
| WD repeat and HMG-box DNA binding protein 1 (WDHD1), transcript variant 1, mRNA [NM_007086] | A_23_P25873 | WDHD1 | 3.07 |
| aryl-hydrocarbon receptor nuclear translocator 2 (ARNT2), mRNA [NM_014862] | A_23_P83579 | ARNT2 | 3.06 |
| angiomotin like 2 (AMOTL2), mRNA [NM_016201] | A_23_P166686 | AMOTL2 | 3.06 |
| myeloid/lymphoid or mixed-lineage leukemia (trithorax homolog, Drosophila); translocated to, 4 (MLLT4), transcript variant 1, mRNA [NM_001040001] | A_23_P436353 | MLLT4 | 3.06 |
| Pentatricopeptide repeat domain 3 (PTCD3), mRNA [NM_017952] | A_23_P154266 | PTCD3 | 3.06 |
| fibroblast growth factor 2 (basic) (FGF2), mRNA [NM_002006] | A_23_P218918 | FGF2 | 3.05 |
| renal tumor antigen (RAGE), mRNA [NM_014226] | A_23_P76731 | RAGE | 3.05 |
| Cbp/p300-interacting transactivator, with Glu/Asp-rich carboxy-terminal domain, 2 (CITED2), mRNA [NM_006079] | A_23_P214969 | CITED2 | 3.05 |
| G patch domain containing 2 (GPATCH2), mRNA [NM_018040] | A_23_P34757 | GPATCH2 | 3.05 |
| actin, gamma 1 (ACTG1), mRNA [NM_001614] | A_23_P44037 | ACTG1 | 3.05 |
| potassium channel tetramerisation domain containing 5 (KCTD5), mRNA [NM_018992] | A_32_P191084 | KCTD5 | 3.05 |
| signal peptidase complex subunit 3 homolog (S. cerevisiae) (SPCS3), mRNA [NM_021928] | A_23_P132936 | SPCS3 | 3.05 |
| protein phosphatase 2, regulatory subunit B, delta isoform (PPP2R2D), transcript variant 1, mRNA [NM_018461] | A_23_P135778 | PPP2R2D | 3.04 |
| cytokine induced apoptosis inhibitor 1 (CIAPIN1), mRNA [NM_020313] | A_23_P88781 | CIAPIN1 | 3.04 |
| CDK5 regulatory subunit associated protein 1 (CDK5RAP1), transcript variant 2, mRNA [NM_016082] | A_23_P257278 | CDK5RAP1 | 3.04 |
| zinc finger protein 212 (ZNF212), mRNA [NM_012256] | A_23_P415558 | ZNF212 | 3.03 |
| leucine zipper-EF-hand containing transmembrane protein 1 (LETM1), mRNA [NM_012318] | A_23_P94998 | LETM1 | 3.03 |
| bromodomain adjacent to zinc finger domain, 1A (BAZ1A), transcript variant 1, mRNA [NM_013448] | A_23_P76799 | BAZ1A | 3.03 |
| Cytochrome c oxidase subunit 1 (EC 1.9.3.1) (Cytochrome c oxidase polypeptide I). [Source:Uniprot/SWISSPROT;Acc:P00395] [ENST00000361624] | A_23_P301925 | COX1 | 3.02 |
| mohawk homeobox (MKX), mRNA [NM_173576] | A_24_P326491 | MKX | 3.01 |

d-value = significance analysis of microarrays (SAM) t-statistic;
