# Supplementary material for: Sphingosine 1-Phosphate Induces Differentiation of Mesoangioblasts towards Smooth Muscle. A Role for GATA6
Source: PLoS One. 2011 May 24;6(5):e20389. doi: 10.1371/journal.pone.0020389 (PMC3101247; doi:10.1371/journal.pone.0020389)
Supplement: Table S2 — Genes down-regulated by S1P treatment in human mesoangioblasts (DOC) [file pone.0020389.s002.doc]

**"SUPPLEMENTAL MATERIAL."**

**Table S2** Genes down-regulated by S1P treatment in human mesoangioblasts

**GEO accession number GSE17596**[**http://www.ncbi.nlm.nih.gov/geo/query/acc.cgi?token=tpivteiccuwkspi&acc=GSE17596**](http://www.ncbi.nlm.nih.gov/geo/query/acc.cgi?token=tpivteiccuwkspi&acc=GSE17596)

| Gene Description | Agilent ID | Gene Names | d-value |
| --- | --- | --- | --- |
| aldo-keto reductase family 1, member B10 (aldose reductase) (AKR1B10), mRNA [NM_020299] | A_23_P93641 | AKR1B10 | -10.05 |
| phorbol-12-myristate-13-acetate-induced protein 1 (PMAIP1), mRNA [NM_021127] | A_23_P207999 | PMAIP1 | -7.47 |
| apolipoprotein B mRNA editing enzyme, catalytic polypeptide-like 3C (APOBEC3C), mRNA [NM_014508] | A_23_P120931 | APOBEC3C | -6.52 |
| AA495894 zw04g12.r1 Soares_NhHMPu_S1 cDNA clone IMAGE:768358 5' similar to contains Alu repetitive element;, mRNA sequence [AA495894] | A_32_P193166 | AA495894 | -6.29 |
| pleckstrin homology-like domain, family A, member 1 (PHLDA1), mRNA [NM_007350] | A_24_P915692 | PHLDA1 | -6.18 |
| chromosome 14 open reading frame 139, mRNA (cDNA clone MGC:14946 IMAGE:3532035), complete cds. [BC008299] | A_23_P14302 | C14orf139 | -6.17 |
| pleckstrin homology-like domain, family A, member 1 (PHLDA1), mRNA [NM_007350] | A_23_P76450 | PHLDA1 | -6.00 |
| phorbol-12-myristate-13-acetate-induced protein 1 (PMAIP1), mRNA [NM_021127] | A_23_P207999 | PMAIP1 | -5.89 |
| phorbol-12-myristate-13-acetate-induced protein 1 (PMAIP1), mRNA [NM_021127] | A_23_P207999 | PMAIP1 | -5.78 |
| ST3 beta-galactoside alpha-2,3-sialyltransferase 5 (ST3GAL5), transcript variant 1, mRNA [NM_003896] | A_23_P136573 | ST3GAL5 | -5.60 |
| GLIS family zinc finger 1 (GLIS1), mRNA [NM_147193] | A_23_P316612 | GLIS1 | -5.50 |
| phorbol-12-myristate-13-acetate-induced protein 1 (PMAIP1), mRNA [NM_021127] | A_23_P207999 | PMAIP1 | -5.41 |
| BCL2 binding component 3 (BBC3), mRNA [NM_014417] | A_24_P305312 | BBC3 | -5.38 |
| aquaporin 1 (Colton blood group) (AQP1), mRNA [NM_198098] | A_23_P19894 | AQP1 | -5.23 |
| hypothetical protein LOC374920 (LOC374920), mRNA [NM_199341] | A_23_P4922 | LOC374920 | -5.23 |
| hypothetical protein LOC284352, mRNA (cDNA clone IMAGE:4779950), with apparent retained intron. [BC039061] | A_23_P101308 | ENST00000221462 | -5.22 |
| heme oxygenase (decycling) 1 (HMOX1), mRNA [NM_002133] | A_23_P120883 | HMOX1 | -5.19 |
| tetraspanin 14 (TSPAN14), mRNA [NM_030927] | A_24_P98613 | TSPAN14 | -4.99 |
| regulator of G-protein signalling 17 (RGS17), mRNA [NM_012419] | A_23_P156861 | RGS17 | -4.99 |
| glycine dehydrogenase (decarboxylating) (GLDC), mRNA [NM_000170] | A_23_P123596 | GLDC | -4.98 |
| trichorhinophalangeal syndrome I (TRPS1), mRNA [NM_014112] | A_23_P134755 | TRPS1 | -4.87 |
| phorbol-12-myristate-13-acetate-induced protein 1 (PMAIP1), mRNA [NM_021127] | A_23_P207999 | PMAIP1 | -4.86 |
| Unknown | A_24_P485742 | THC2689427 | -4.85 |
| symplekin (SYMPK), mRNA [NM_004819] | A_23_P78808 | SYMPK | -4.76 |
| ALU7_HUMAN (P39194) Alu subfamily SQ sequence contamination warning entry, partial (8%) [THC2541992] | A_32_P199824 | THC2541992 | -4.74 |
| Q3WYF8_9ACTN (Q3WYF8) Sulfotransferase, partial (6%) [THC2671898] | A_23_P152570 | THC2671898 | -4.74 |
| mannosidase, alpha, class 1A, member 1 (MAN1A1), mRNA [NM_005907] | A_23_P156425 | MAN1A1 | -4.74 |
| RAP2B, member of RAS oncogene family (RAP2B), mRNA [NM_002886] | A_24_P123052 | RAP2B | -4.71 |
| aldo-keto reductase family 1, member B10 (aldose reductase) (AKR1B10), mRNA [NM_020299] | A_24_P129341 | AKR1B10 | -4.69 |
| SRY (sex determining region Y)-box 4 (SOX4), mRNA [NM_003107] | A_23_P82169 | SOX4 | -4.65 |
| transducin-like enhancer of split 1 (E(sp1) homolog, Drosophila) (TLE1), mRNA [NM_005077] | A_23_P135239 | TLE1 | -4.62 |
| T-box 3 (ulnar mammary syndrome) (TBX3), transcript variant 2, mRNA [NM_016569] | A_23_P383819 | TBX3 | -4.61 |
| catenin, beta interacting protein 1 (CTNNBIP1), transcript variant 1, mRNA [NM_020248] | A_23_P23584 | CTNNBIP1 | -4.60 |
| integral membrane protein 2C (ITM2C), transcript variant 1, mRNA [NM_030926] | A_24_P379820 | ITM2C | -4.59 |
| v-maf musculoaponeurotic fibrosarcoma oncogene homolog (avian) (MAF), transcript variant 2, mRNA [NM_001031804] | A_23_P397376 | MAF | -4.58 |
| ST3 beta-galactoside alpha-2,3-sialyltransferase 5 (ST3GAL5), transcript variant 1, mRNA [NM_003896] | A_23_P311869 | ST3GAL5 | -4.56 |
| MAF1 homolog (S. cerevisiae) (MAF1), mRNA [NM_032272] | A_23_P33072 | MAF1 | -4.56 |
| phorbol-12-myristate-13-acetate-induced protein 1 (PMAIP1), mRNA [NM_021127] | A_23_P207999 | PMAIP1 | -4.53 |
| tripartite motif-containing 63 (TRIM63), mRNA [NM_032588] | A_23_P114983 | TRIM63 | -4.53 |
| Unknown | A_32_P194563 | THC2651751 | -4.50 |
| retinoic acid induced 2 (RAI2), mRNA [NM_021785] | A_23_P254165 | RAI2 | -4.48 |
| ubiquitin-like 3 (UBL3), mRNA [NM_007106] | A_24_P330234 | UBL3 | -4.48 |
| phorbol-12-myristate-13-acetate-induced protein 1 (PMAIP1), mRNA [NM_021127] | A_23_P207999 | PMAIP1 | -4.46 |
| Unknown | A_24_P246825 | THC2677796 | -4.42 |
| cornichon homolog 3 (Drosophila) (CNIH3), mRNA [NM_152495] | A_23_P384044 | CNIH3 | -4.41 |
| transient receptor potential cation channel, subfamily C, member 1 (TRPC1), mRNA [NM_003304] | A_24_P28977 | TRPC1 | -4.39 |
| suppression of tumorigenicity 13 (colon carcinoma) (Hsp70 interacting protein) (ST13), mRNA [NM_003932] | A_23_P68949 | ST13 | -4.34 |
| phorbol-12-myristate-13-acetate-induced protein 1 (PMAIP1), mRNA [NM_021127] | A_23_P207999 | PMAIP1 | -4.33 |
| transmembrane protein 77 (TMEM77), mRNA [NM_178454] | A_23_P74550 | TMEM77 | -4.33 |
| chemokine (C-C motif) receptor 1 (CCR1), mRNA [NM_001295] | A_24_P148717 | CCR1 | -4.31 |
| Unknown | A_24_P93462 | A_24_P93462 | -4.29 |
| CD86 molecule (CD86), transcript variant 2, mRNA [NM_006889] | A_24_P131589 | CD86 | -4.28 |
| zinc finger protein 616, mRNA (cDNA clone MGC:45556 IMAGE:4186857), complete cds. [BC032805] | A_32_P186921 | ZNF616 | -4.28 |
| PREDICTED: hypothetical protein LOC92312 (LOC92312), mRNA [XM_044166] | A_32_P96036 | LOC92312 | -4.27 |
| endothelin receptor type A (EDNRA), mRNA [NM_001957] | A_24_P217572 | EDNRA | -4.26 |
| 602285795F1 NIH_MGC_86 cDNA clone IMAGE:4373207 5', mRNA sequence [BG114486] | A_32_P2354 | BG114486 | -4.25 |
| early B-cell factor 3 (EBF3), mRNA [NM_001005463] | A_23_P381645 | EBF3 | -4.22 |
| v-maf musculoaponeurotic fibrosarcoma oncogene homolog B (avian) (MAFB), mRNA [NM_005461] | A_23_P17345 | MAFB | -4.20 |
| protein kinase C, epsilon (PRKCE), mRNA [NM_005400] | A_23_P250564 | PRKCE | -4.16 |
| HMBX1_HUMAN (Q6NT76) Homeobox-containing protein 1, partial (39%) [THC2609820] | A_24_P932736 | THC2609820 | -4.15 |
| ubiquitin-like 3 (UBL3), mRNA [NM_007106] | A_23_P140029 | UBL3 | -4.14 |
| protein-L-isoaspartate (D-aspartate) O-methyltransferase domain containing 1 (PCMTD1), mRNA [NM_052937] | A_24_P110967 | PCMTD1 | -4.12 |
| bromodomain and WD repeat domain containing 1 (BRWD1), transcript variant 1, mRNA [NM_018963] | A_23_P211136 | BRWD1 | -4.11 |
| ring finger protein 144 (RNF144), mRNA [NM_014746] | A_24_P37264 | RNF144 | -4.09 |
| growth arrest-specific 1 (GAS1), mRNA [NM_002048] | A_23_P83134 | GAS1 | -4.09 |
| fuzzy homolog (Drosophila) (FUZ), mRNA [NM_025129] | A_23_P373568 | FUZ | -4.08 |
| CKLF-like MARVEL transmembrane domain containing 8 (CMTM8), mRNA [NM_178868] | A_23_P40880 | CMTM8 | -4.06 |
| Pre-B-cell leukemia transcription factor 1 (Homeobox protein PBX1) (Homeobox protein PRL). [Source:Uniprot/SWISSPROT;Acc:P40424] [ENST00000328681] | A_32_P25397 | PBX1 | -4.03 |
| clone 24525 mRNA sequence. [AF070529] | A_32_P3113 | AF070529 | -4.03 |
| FK506 binding protein 10, 65 kDa (FKBP10), mRNA [NM_021939] | A_23_P15727 | FKBP10 | -4.02 |
| E2F transcription factor 7 (E2F7), mRNA [NM_203394] | A_32_P210202 | E2F7 | -4.01 |
| MBC3205 (UNQ501), mRNA [NM_198536] | A_23_P90099 | UNQ501 | -4.01 |
| neuritin 1 (NRN1), mRNA [NM_016588] | A_23_P414519 | NRN1 | -4.01 |
| R-spondin 2 homolog (Xenopus laevis) (RSPO2), mRNA [NM_178565] | A_23_P430930 | RSPO2 | -4.01 |
| GLI-Kruppel family member GLI3 (Greig cephalopolysyndactyly syndrome) (GLI3), mRNA [NM_000168] | A_23_P111531 | GLI3 | -4.00 |
| inhibitor of DNA binding 4, dominant negative helix-loop-helix protein (ID4), mRNA [NM_001546] | A_23_P59375 | ID4 | -4.00 |
| NISC_gl03c01.x1 NCI_CGAP_Lei2 cDNA clone IMAGE:3289728 3', mRNA sequence [CB051804] | A_24_P347418 | CB051804 | -3.98 |
| forkhead box Q1 (FOXQ1), mRNA [NM_033260] | A_32_P164246 | FOXQ1 | -3.97 |
| KRR1, small subunit (SSU) processome component, homolog (yeast) (KRR1), mRNA [NM_007043] | A_23_P2216 | KRR1 | -3.97 |
| jumonji domain containing 1C (JMJD1C), transcript variant 2, mRNA [NM_004241] | A_23_P300301 | JMJD1C | -3.97 |
| chromosome 20 open reading frame 39 (C20orf39), mRNA [NM_024893] | A_23_P251043 | C20orf39 | -3.94 |
| ephrin-B1 (EFNB1), mRNA [NM_004429] | A_24_P365807 | EFNB1 | -3.92 |
| heat shock 70kDa protein 4-like (HSPA4L), mRNA [NM_014278] | A_23_P363936 | HSPA4L | -3.90 |
| Homo sapiens, clone IMAGE:5166482, mRNA, partial cds. [BC028192] | A_32_P68408 | LOC161635 | -3.90 |
| wingless-type MMTV integration site family, member 3 (WNT3), mRNA [NM_030753] | A_23_P130158 | WNT3 | -3.89 |
| nuclear receptor subfamily 0, group B, member 1 (NR0B1), mRNA [NM_000475] | A_23_P73632 | NR0B1 | -3.88 |
| leucine rich repeat containing 62 (LRRC62), mRNA [NM_052906] | A_32_P219279 | LRRC62 | -3.85 |
| mRNA for KIAA1063 protein, partial cds. [AB028986] | A_24_P94034 | USP22 | -3.82 |
| myosin binding protein H (MYBPH), mRNA [NM_004997] | A_23_P148737 | MYBPH | -3.81 |
| guanine nucleotide binding protein (G protein), gamma 2 (GNG2), mRNA [NM_053064] | A_32_P208403 | GNG2 | -3.80 |
| dishevelled, dsh homolog 2 (Drosophila) (DVL2), mRNA [NM_004422] | A_23_P55342 | DVL2 | -3.80 |
| zinc finger protein 521 (ZNF521), mRNA [NM_015461] | A_23_P159027 | ZNF521 | -3.79 |
| DNA-damage-inducible transcript 4-like (DDIT4L), mRNA [NM_145244] | A_23_P302672 | DDIT4L | -3.79 |
| SRY (sex determining region Y)-box 4 (SOX4), mRNA [NM_003107] | A_24_P911676 | SOX4 | -3.78 |
| Rho GTPase activating protein 22 (ARHGAP22), mRNA [NM_021226] | A_23_P75310 | ARHGAP22 | -3.78 |
| bromodomain and WD repeat domain containing 1 (BRWD1), transcript variant 1, mRNA [NM_018963] | A_24_P190541 | BRWD1 | -3.78 |
| similar to 40S ribosomal protein S3a (V-fos transformation effector protein) (LOC391706), mRNA [Source:RefSeq_dna;Acc:XR_018587] [ENST00000389400] | A_24_P675947 | ENST00000389400 | -3.76 |
| Unknown | A_32_P229447 | A_32_P229447 | -3.75 |
| coiled-coil domain containing 98 (CCDC98), mRNA [NM_139076] | A_23_P253464 | CCDC98 | -3.73 |
| AW946823 RC2-ET0022-080500-012-b10 ET0022 cDNA, mRNA sequence [AW946823] | A_32_P96752 | AW946823 | -3.73 |
| chromosome 12 open reading frame 35 (C12orf35), mRNA [NM_018169] | A_23_P98930 | C12orf35 | -3.71 |
| tumor necrosis factor receptor superfamily, member 21 (TNFRSF21), mRNA [NM_014452] | A_23_P30666 | TNFRSF21 | -3.70 |
| C-type lectin domain family 2, member B (CLEC2B), mRNA [NM_005127] | A_24_P201702 | CLEC2B | -3.69 |
| RALBP1 associated Eps domain containing 1 (REPS1), mRNA [NM_031922] | A_23_P70748 | REPS1 | -3.69 |
| chromosome 22 open reading frame 13 (C22orf13), mRNA [NM_031444] | A_23_P17773 | C22orf13 | -3.69 |
| short stature homeobox 2 (SHOX2), transcript variant SHOX2a, mRNA [NM_006884] | A_23_P124384 | SHOX2 | -3.69 |
| chromosome 20 open reading frame 42 (C20orf42), mRNA [NM_017671] | A_23_P131935 | C20orf42 | -3.68 |
| Q3MHD6_HUMAN (Q3MHD6) COX17 homolog, cytochrome c oxidase assembly protein, partial (98%) [THC2671344] | A_23_P144244 | THC2671344 | -3.68 |
| RAB42, member RAS oncogene family (RAB42), mRNA [NM_152304] | A_23_P434919 | RAB42 | -3.67 |
| membrane protein, palmitoylated 1, 55kDa (MPP1), mRNA [NM_002436] | A_23_P171296 | MPP1 | -3.67 |
| BX419129 FETAL BRAIN cDNA clone CS0DF013YC22 5-PRIME, mRNA sequence [BX419129] | A_32_P40999 | BX419129 | -3.67 |
| prostaglandin F receptor (FP) (PTGFR), transcript variant 2, mRNA [NM_001039585] | A_24_P302172 | PTGFR | -3.65 |
| chromosome 3 open reading frame 34 (C3orf34), mRNA [NM_032898] | A_32_P212058 | C3orf34 | -3.65 |
| centrin, EF-hand protein, 2 (CETN2), mRNA [NM_004344] | A_23_P73493 | CETN2 | -3.64 |
| vacuolar protein sorting 37 homolog D (S. cerevisiae) (VPS37D), mRNA [NM_001077621] | A_23_P409417 | VPS37D | -3.64 |
| iroquois homeobox protein 5 (IRX5), mRNA [NM_005853] | A_23_P9779 | IRX5 | -3.62 |
| Rho GTPase activating protein 18 (ARHGAP18), mRNA [NM_033515] | A_32_P162250 | ARHGAP18 | -3.62 |
| Unknown | A_32_P203939 | THC2689427 | -3.62 |
| HCLS1 binding protein 3 (HS1BP3), mRNA [NM_022460] | A_24_P139943 | HS1BP3 | -3.61 |
| solute carrier family 25 (mitochondrial carrier; phosphate carrier), member 23 (SLC25A23), mRNA [NM_024103] | A_23_P208450 | SLC25A23 | -3.58 |
| signal-induced proliferation-associated 1 like 2 (SIPA1L2), mRNA [NM_020808] | A_23_P137470 | SIPA1L2 | -3.57 |
| ribosomal protein S2 (RPS2), mRNA [NM_002952] | A_32_P14544 | RPS2 | -3.57 |
| peroxisome proliferator-activated receptor gamma (PPARG), transcript variant 3, mRNA [NM_138711] | A_23_P252062 | PPARG | -3.57 |
| Unknown | A_32_P199506 | THC2662749 | -3.56 |
| AA599881 ag32e07.s1 Human bone marrow stromal cells cDNA clone IMAGE:1091268 3' similar to gb:M21574 ALPHA PLATELET-DERIVED GROWTH FACTOR RECEPTOR PRECURSOR (HUMAN);, mRNA sequence [AA599881] | A_32_P100379 | PDGFRA | -3.55 |
| RPA interacting protein (RPAIN), transcript variant 2, mRNA [NM_032308] | A_23_P66766 | RPAIN | -3.54 |
| CD109 molecule (CD109), mRNA [NM_133493] | A_23_P331928 | CD109 | -3.54 |
| replication protein A4, 34kDa (RPA4), mRNA [NM_013347] | A_23_P254212 | RPA4 | -3.54 |
| bromodomain containing 3 (BRD3), mRNA [NM_007371] | A_23_P216689 | BRD3 | -3.54 |
| ADP-ribosylation factor-like 6 interacting protein 1 (ARL6IP1), mRNA [NM_015161] | A_24_P356218 | ARL6IP1 | -3.52 |
| zinc finger protein 106 homolog (mouse) (ZFP106), mRNA [NM_022473] | A_23_P77310 | ZFP106 | -3.51 |
| PREDICTED: similar to heat shock 70kD protein binding protein (LOC729992), mRNA [XR_016071] | A_24_P315457 | ENST00000323501 | -3.51 |
| chromosome 9 open reading frame 23 (C9orf23), transcript variant 2, mRNA [NM_148179] | A_23_P169409 | C9orf23 | -3.51 |
| SLC2A4 regulator (SLC2A4RG), mRNA [NM_020062] | A_24_P365442 | SLC2A4RG | -3.50 |
| PREDICTED: hypothetical LOC391540 (LOC391540), mRNA [XR_017295] | A_24_P15610 | LOC391540 | -3.49 |
| sprouty-related, EVH1 domain containing 1 (SPRED1), mRNA [NM_152594] | A_23_P54460 | SPRED1 | -3.48 |
| phorbol-12-myristate-13-acetate-induced protein 1 (PMAIP1), mRNA [NM_021127] | A_23_P207999 | PMAIP1 | -3.47 |
| nuclear receptor subfamily 1, group D, member 1 (NR1D1), mRNA [NM_021724] | A_23_P420873 | NR1D1 | -3.46 |
| AGENCOURT_6447766 NIH_MGC_92 cDNA clone IMAGE:5587170 5', mRNA sequence [BM468849] | A_32_P107752 | BM468849 | -3.45 |
| pleckstrin homology-like domain, family A, member 1 (PHLDA1), mRNA [NM_007350] | A_24_P943597 | PHLDA1 | -3.45 |
| sestrin 1 (SESN1), mRNA [NM_014454] | A_24_P346339 | SESN1 | -3.45 |
| chromosome 1 open reading frame 38, mRNA (cDNA clone IMAGE:5170350), complete cds. [BC031655] | A_23_P873 | C1orf38 | -3.45 |
| paternally expressed 10 (PEG10), transcript variant 1, mRNA [NM_001040152] | A_23_P82503 | PEG10 | -3.45 |
| protein phosphatase 2 (formerly 2A), regulatory subunit B'', alpha (PPP2R3A), transcript variant 1, mRNA [NM_002718] | A_23_P10401 | PPP2R3A | -3.44 |
| potassium channel tetramerisation domain containing 17 (KCTD17), mRNA [NM_024681] | A_24_P108779 | KCTD17 | -3.44 |
| likely ortholog of mouse neighbor of Punc E11 (NOPE), mRNA [NM_020962] | A_23_P14673 | NOPE | -3.44 |
| zinc finger, FYVE domain containing 19 (ZFYVE19), mRNA [NM_001077268] | A_23_P65930 | ZFYVE19 | -3.42 |
| proliferation-inducing protein 9 mRNA, complete cds. [AY239294] | A_32_P194874 | AY239294 | -3.42 |
| cDNA FLJ45609 fis, clone BRTHA3023590. [AK127516] | A_24_P687326 | C9orf109 | -3.41 |
| Tetraspanin-14 (Tspan-14) (Transmembrane 4 superfamily member 14) (DC- TM4F2). [Source:Uniprot/SWISSPROT;Acc:Q8NG11] [ENST00000265450] | A_32_P91902 | TSPAN14 | -3.41 |
| chromosome 5 open reading frame 33 (C5orf33), mRNA [NM_153013] | A_23_P337168 | C5orf33 | -3.40 |
| alanyl (membrane) aminopeptidase (aminopeptidase N, aminopeptidase M, microsomal aminopeptidase, CD13, p150) (ANPEP), mRNA [NM_001150] | A_23_P88626 | ANPEP | -3.39 |
| transketolase (Wernicke-Korsakoff syndrome) (TKT), mRNA [NM_001064] | A_23_P92082 | TKT | -3.38 |
| transcription elongation factor A (SII)-like 8 (TCEAL8), transcript variant 1, mRNA [NM_153333] | A_23_P11331 | TCEAL8 | -3.37 |
| ABI gene family, member 3 (NESH) binding protein (ABI3BP), mRNA [NM_015429] | A_23_P218858 | ABI3BP | -3.35 |
| pleckstrin homology-like domain, family A, member 1 (PHLDA1), mRNA [NM_007350] | A_23_P338912 | PHLDA1 | -3.35 |
| Unknown | A_32_P112078 | THC2538534 | -3.35 |
| cDNA FLJ11918 fis, clone HEMBB1000272. [AK021980] | A_24_P931428 | AK021980 | -3.35 |
| remodeling and spacing factor 1 (RSF1), mRNA [NM_016578] | A_24_P246717 | RSF1 | -3.34 |
| upregulated during skeletal muscle growth 5 homolog (mouse) (USMG5), mRNA [NM_032747] | A_23_P127095 | USMG5 | -3.33 |
| FERM domain containing 4A (FRMD4A), mRNA [NM_018027] | A_24_P43959 | FRMD4A | -3.33 |
| 602664870F1 NIH_MGC_60 cDNA clone IMAGE:4804769 5', mRNA sequence [BG777521] | A_24_P647163 | BG777521 | -3.33 |
| spermatogenesis associated 6 [Source:RefSeq_peptide;Acc:NP_061946] [ENST00000371847] | A_23_P23705 | SPATA6 | -3.33 |
| BQ374929 MR1-TN0045-060900-005-d11 TN0045 cDNA, mRNA sequence [BQ374929] | A_32_P100076 | BQ374929 | -3.32 |
| EF-hand domain family, member B (EFHB), mRNA [NM_144715] | A_23_P417261 | EFHB | -3.32 |
| calcium modulating ligand (CAMLG), mRNA [NM_001745] | A_23_P213728 | CAMLG | -3.32 |

d-value = significance analysis of microarrays (SAM) t-statistic;
